# Tuberculosis/cryptococcosis co-infection in China between 1965 and 2016

**DOI:** 10.1038/emi.2017.61

**Published:** 2017-08-23

**Authors:** Wenjie Fang, Lei Zhang, Jia Liu, David W Denning, Ferry Hagen, Weiwei Jiang, Nan Hong, Shuwen Deng, Xia Lei, Danqi Deng, Wanqing Liao, Jianping Xu, Teun Boekhout, Min Chen, Weihua Pan

**Affiliations:** 1 Department of Dermatology, Shanghai Key Laboratory of Molecular Medical Mycology, Shanghai Institute of Medical Mycology, Changzheng Hospital, Second Military Medical University, Shanghai 200003, China; 2 Department of Dermatology, Changzheng Hospital, Second Military Medical University, Shanghai 200003, China; 3 Westerdijk Fungal Biodiversity Institute, Utrecht 3584, The Netherlands; 4 National Aspergillosis Centre, University Hospital of South Manchester and University of Manchester, Manchester M13 9PL, UK; 5 Department of Medical Microbiology and Infectious Diseases, Canisius-Wilhelmina Hospital, Nijmegen 6532, The Netherlands; 6 Department of Dermatology, Daping Hospital, Third Military Medical University, Chongqing 400042, China; 7 Department of Dermatology, The Second Affiliated Hospital of Kunming Medical University, Kunming 650504, China; 8 Department of Biology, McMaster University, Hamilton, Ontario L8S 4M1, Canada; 9 Institute of Biodiversity and Ecosystem Dynamics, University of Amsterdam, Amsterdam 1012, The Netherlands

**Keywords:** China, co-infection, cryptococcosis, tuberculosis

## Abstract

Cases of tuberculosis/cryptococcosis co-infection are rapidly increasing in China. However, most studies addressing this co-infection have been published in Chinese journals, and this publication strategy has obscured this disease trend for scientists in other parts of the world. Our investigation found that 62.9% of all co-infection cases worldwide were reported in the Chinese population (*n*=197) between 1965 and 2016, and 56.3% of these Chinese cases were reported after 2010. Nearly all cases originated from the warm and wet monsoon regions of China. HIV-positive subjects tended to correlate with more severe manifestations of a tuberculosis/cryptococcosis co-infection than those without HIV. Notablely, dual tubercular/cryptococcal meningitis was the most frequent (54.0%) and most easily misdiagnosed (95.2%, *n*=40/42) co-infection. We also found that the combined use of cerebrospinal fluid pressure and concentrations of glucose, protein and chlorine might be an inexpensive and effective indicator to differentiate tubercular/cryptococcal co-infection meningitis from tubercular meningitis and cryptococcal meningitis.

## INTRODUCTION

*Mycobacterium tuberculosis* (TB), first isolated by the German researcher Robert Koch, has been the cause of a deadly global epidemic for more than a century.^1^ Although the global morbidity and mortality rates of TB have steadily decreased since the early twentieth century, nearly 10.4 million people were newly infected and 1.4 million people died from TB worldwide in 2015.^2, 3, 4^ China has the third largest TB burden after India and Indonesia, constituting 10% of all cases worldwide.^3, 5^ Since the 1990s, sustained nationwide attention has been paid to the surveillance and control of TB in China. The prevalence of TB in China has steadily decreased from 170 to 59 cases per 100 000 people over the last 20 years primarily because of the use of improved early diagnostic tools and the increased implementation of directly observed treatment, short-course (DOTS) strategy.^6^ However, the re-emergence of this disease and its associated challenges regarding the control of other pandemic pathogen co-infections (for example, HIV, Ebola virus, hepatitis B virus and various parasites) has led to increased global concern.^7, 8, 9, 10, 11, 12^

Cryptococcosis is an emerging yeast infection caused by strains of the *Cryptococcus neoformans* and *Cryptococcus gattii* species complexes.^13^ These pathogenic *Cryptococcus* species affect patients with immuno-compromising conditions such as HIV infection and individuals who are apparently healthy.^14, 15, 16, 17^ A global estimation suggested that each year ~960 000 new cases of cryptococcal meningitis occur in the HIV-positive patient population, leading to ~620 000 deaths within 90 days.^16^ Our previous studies revealed unique features of the molecular epidemiology and clinical profiles of cryptococcosis in China, including strain genotype homogeneity and the involvement of a limited number of patients with HIV infection.^18, 19^ By contrast, in other countries and regions, cryptococcosis was caused by strains with relatively high genotypic heterogeneity, and it dominated among patients suffering from a HIV infection. Given such unique features of cryptococcosis in China, we were interested in whether other immune-compromising infections might be associated with cryptococcosis in the Chinese population.

Studies have confirmed the synergistic growth-promoting association and high similarity in pathogenic processes between fungal infections and TB.^20, 21^ However, even though sporadic TB/cryptococcosis co-infection cases have been published in international journals, relatively limited information is available to the international infectious diseases community on TB and cryptococcosis co-infection in China. This lack of attention was not due to the lack of reports but because most of these cases were published in Chinese journals that are not easily accessible to researchers outside China. Indeed, the first case of TB/cryptococcosis co-infection in China was diagnosed more than half a century ago, and similar cases have emerged with increasing frequency over recent decades in China. Recently, we described seven TB/cryptococcosis co-infection cases from the literature and a recent case from Shanghai, China.^22^ The objectives of this study are to perform a systematic literature review and conduct a comparative analysis of various hospital cases spanning the past 50 years to investigate the epidemiological and clinical profile of TB/cryptococcosis co-infection in China.

## MATERIALS AND METHODS

### Study registration and ethical approval

This study strictly followed the reporting guidelines of the Preferred Reporting Items for Systematic Reviews and Meta-Analyses (PRISMA) statement; the registration number of the study protocol in PROSPERO is CRD42016039341. The Ethics Committee of Changzheng Hospital, Shanghai, China, approved the use of the clinical data from etiologically confirmed cases of tubercular meningitis and cryptococcal meningitis for a comparative analysis (approval number: 2016SL021).

### Search strategies and inclusion criteria

We searched PubMed, Embase, the China National Knowledge Infrastructure (CNKI), the Chinese Biomedical Literature Service System (SinoMed) and WanFang database for international or local studies published in Chinese journals. The following strategy represents an example for a PubMed retrieval that was not restricted by language, publication date or study type:

(Tuberculosis(Mesh) OR ‘Mycobacterium Infections’(Mesh) OR Tuberculoma(Mesh) OR ‘king’s evil’(Mesh) OR ‘Erythema Induratum’(Mesh) OR ‘Lupus Vulgaris’(Mesh) OR ‘Empyema, Tuberculous’(Mesh) OR Silicotuberculosis(Mesh) OR TB OR Mycobacterium OR MTB OR mycobacteria OR ‘white plague’ OR phthisis OR PTB OR decline OR tubercular OR tuberculoderma OR tuberculoma OR scrofuloderma OR silicotuberculosis OR ‘king’s evil’ OR ‘Erythema Induratum’ OR ‘Lupus Vulgaris’ OR ‘Empyema, Tuberculous’) AND (Cryptococcosis(Mesh) OR ‘Meningitis, Cryptococcal’(Mesh) OR Cryptococcus(Mesh) OR ‘*Cryptococcus neoformans*’(Mesh) OR ‘*Cryptococcus gattii*’(Mesh) OR Filobasidiella OR Cryptococc* OR neoformans OR grubii OR gatti* OR Torul*).

Manual retrieval was performed based on the reference lists of relevant articles. If necessary, the corresponding authors were contacted for additional clinical information on the reported cases. Review articles without original data were excluded. We also checked whether the same case was reported in multiple publications; if so, it was included only once.

The etiological diagnosis of cryptococcosis is based on India ink staining, culture and the latex agglutination test,^23^ whereas the etiological diagnosis of TB is based on acid-fast staining, culture, histological examination and diagnostic PCR.^14, 24^ TB/cryptococcal co-infection was defined as cases in which TB and cryptococcosis co-existed simultaneously and both were diagnosed. Similar to Ascioglu *et al.* and Xiao *et al.*,^25, 26^ we placed a high value on the epidemiological and clinical use of cases with adequate information supporting co-infection, even though they may not have sufficient etiological evidence. Therefore, we included and separately analyzed both etiologically and non-etiologically diagnosed cases of co-infection in our study. The cerebrospinal fluid (CSF) pressure and the concentrations of glucose, protein and chlorine in the CSF of 111 patients with cryptococcal meningitis and 69 patients with tubercular meningitis from Shanghai Changzheng Hospital were included in the comparison analysis.

### Data collection and statistical analyses

Two authors independently extracted the relevant information of each paper, including the geographical origin of the patient. SPSS (version 21, IBM Corp., Armonk, NY, USA) and SigmaPlot (version 12.5, Systat Software, San Jose, CA, USA) were used for the statistical analyses. The categorical variables were compared using the *χ*^2^-test. The Nemenyi test was used as a *post hoc* test. A *P*-value of ⩽0.05 was considered to be statistically significant.

## RESULTS

### Identification of reports and cases

**Figure 1:**
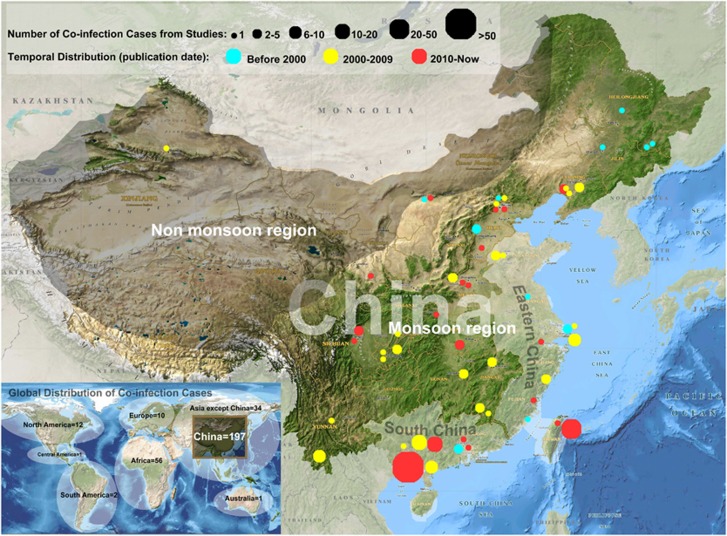
Nationwide distribution of tuberculosis (TB)/cryptococcosis co-infection cases from 1965 to 2016.

Our search strategy identified 197 cases of TB/cryptococcosis co-infection in China in 56 studies, which accounted for 62.9% (*n*=197/313) of all cases worldwide (Supplementary Figure S1). A total of 54 studies (including 173 cases) were written and published in local Chinese journals. All studies reporting non-Chinese cases (not included in our study) and two Chinese studies were published in international journals. A total of 49 (87.5%) studies were conducted at tertiary hospitals in China. Nearly all of the cases (99.5%, *n*=196/197) were collected from the monsoon region of China, particularly from southern and eastern China (71.1%, *n*=140/197; Figure 1). The first case report in the literature described a 31-year-old woman with pulmonary TB and cryptococcal meningitis who was admitted to Beijing Tuberculosis Hospital on 2 July 1964. Before 2000, only 16 co-infection cases (8.1%) were reported, whereas 70 cases (35.5%) were reported during 2000–2009, and 111 cases (56.3%) were reported between 2010 and 2015. In particular, the two largest studies (involving 23 and 52 cases) were reported after 2010.

### Epidemiological and demographic characteristics

The rates of TB/cryptococcosis co-infection among various populations are summarized as follows. The prevalence of co-infection among the TB population was 0.6% (23 co-infection cases/4053 total cases) between 1993 and 2006 in Taiwan. Another study identified three co-infection cases from 31 tubercular meningitis patients (2004–2005, Liaoning). Three studies revealed that the rates of TB/cryptococcosis co-infection in TB/fungal co-infection ranged from 2.7% to 3.8% (*n*=1/31, 2009–2010, Shandong; *n*=2/74, 2002–2004, Guangdong; and *n*=1/26, 2010–2011, Henan). The prevalence of TB/cryptococcosis co-infection among cryptococcosis was 5.4% (*n*=23/425, 1993–2006, Taiwan), and higher rates (6.7%–26.7%) were reported in children with this infection (*n*=1/15, 2006–2011, Henan; *n*=4/15, 1993–2003, Chongqing). The co-infection rate in cryptococcal meningitis patients with and without HIV/AIDS was 42.9% and 15.0%, respectively (*n*=6/14, 2005–2008, Yunnan; *n*=3/20, 1988–2002, Henan).

Employment status was only available for 17 patients, and it included three businessmen, three factory workers, five farmers and six unemployed persons. Epidemiological and demographic details are presented in Supplementary Table S1.

### Clinical profile

The mean age at co-infection diagnosis was 35.0±2.8 years (95% confidence intervals=29.3–40.7), and males predominated the sample (male/female ratio=2.80). Of the 121 patients with known pre-existing conditions, 45 (37.2%) developed at least one comorbid disease. The most common underlying communicable disease, other than TB or cryptococcosis, was HIV/AIDS (82.2%, *n*=37), followed by hepatitis (*n*=3) and syphilis (*n*=3). The underlying non-communicable diseases included type 2 diabetes (*n*=9), renal diseases (*n*=2) and systemic lupus erythematosus (*n*=2). A total of 26 patients had medical histories of previous TB infection, and another four otherwise healthy patients had been in close contact with patients with acute TB 3 months prior to their onset of TB. No history of cryptococcosis-related pigeon dropping contact was found among the analyzed cases.

Details concerning the affected sites of the dual pathogens were available for 174 cases. Tubercular meningitis and cryptococcal meningitis co-infection was the most frequent combination (*n*=94/174, 54.0%); nine of these patients were also diagnosed with pulmonary TB. Approximately 25.9% (*n*=45/174) of patients were diagnosed with pulmonary TB and pulmonary cryptococcosis co-infection, one of whom was also diagnosed with skin cryptococcosis. A total of 17.2% (*n*=30/174) of patients were diagnosed with pulmonary TB and cryptococcal meningitis.

The most common clinical manifestations of co-infection are summarized in Table 1 (see Supplementary Table S2 for details). Comparative studies between patients with or without HIV were conducted among all cases and especially among etiologically diagnosed cases. Data from all cases showed that TB/cryptococcosis co-infection cases with HIV infections as an underlying condition may have more frequent clinical presentations of weakness, weight loss, cough, sputum, chest pain and papilledema than patients without HIV infection (*P*<0.05). However, these findings were not totally consistent with the findings from etiologically diagnosed cases. Considering the small sample size of the etiologically diagnosed cases, further larger-scale studies should be performed.

### Differential diagnosis

**Figure 2:**
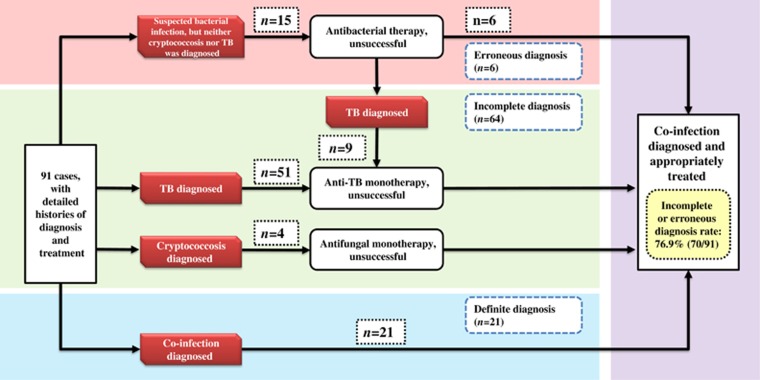
Diagnostic and treatment profiles of tuberculosis (TB)/cryptococcosis co-infection cases.

The detailed histories of diagnosis and treatment were available for 91 cases. Diagnoses were labeled ‘incomplete’ when physicians only considered a single pathogen of the co-infection according to the text of the included studies. Similarly, the diagnoses were labeled ‘erroneous’ when other infections or non-communicable diseases (other than TB or cryptococcosis) were suspected as causes of illness. Careful reading of the included studies revealed that 76.9% (*n*=70/91) of the patients experienced incomplete or erroneous diagnoses (Figure 2). Incomplete diagnoses occurred more frequently (*n*=64/70) than erroneous diagnoses (*n*=15/70). Most incompletely diagnosed individuals were treated as having a TB mono-infection, ignoring the co-existence of cryptococcosis (93.8%, *n*=60/64). Regarding the affected site, 95.2% (*n*=40/42) of the patients with tubercular/cryptococcal meningitis were misdiagnosed, whereas 92.5% (*n*=37/40) of these misdiagnoses were incompletely diagnosed as having tubercular meningitis. However, the misdiagnosis rate was much lower among patients with TB/cryptococcal pneumonia (45.9%, *n*=17/37).

**Figure 3:**
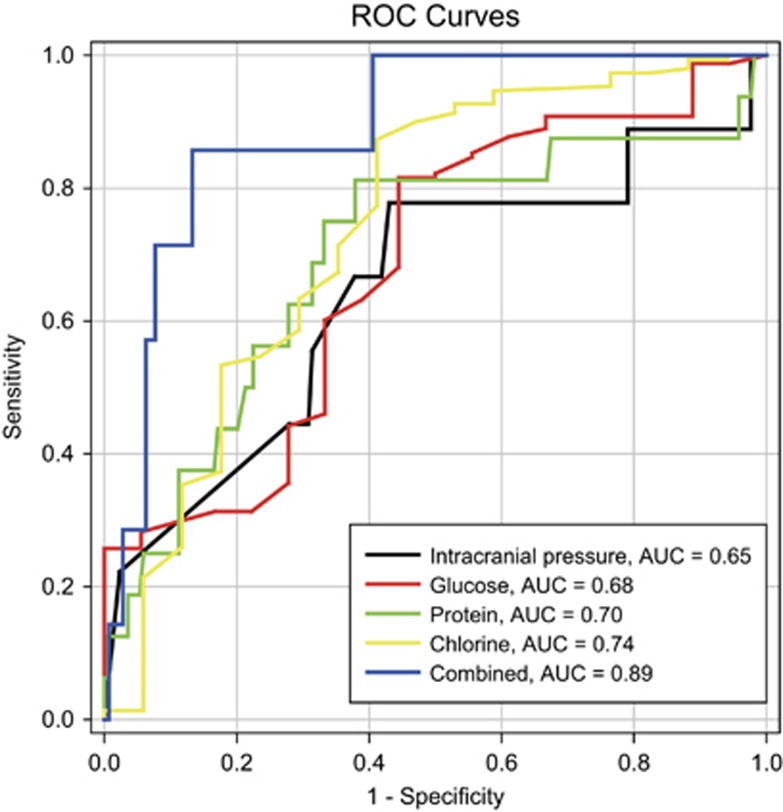
Receiver operating characteristic (ROC) curve area comparison between intracranial pressure, glucose, protein, chlorine and the combined use of the above values.

Because of the high misdiagnosis rate associated with patients with tubercular/cryptococcal meningitis, a comparative analysis among the cases of tubercular/cryptococcal meningitis, cryptococcal meningitis and tubercular meningitis was performed. These comparative studies were also conducted among all cases and especially among etiologically diagnosed cases. A univariate analysis revealed that CSF pressure and the concentrations of glucose, protein and chloride ion in CSF helped the physicians identify meningitis (see Table 2 and Supplementary Tables S3–S5 for details of each group). A particularly high CSF pressure (>266 mm H_2_O) might indicate the existence of *Cryptococcus* (with or without TB). Very-high-CSF protein (>1035.6 mg/L) and low CSF chloride (<119.2 mmol/L) might be related to *M. tuberculosis* infection (with or without cryptococcosis). Moreover, very-low-CSF glucose (<1.9 mmol/L) might be helpful to distinguish co-infection from mono-infection (either TB or cryptococcosis). The above etiologically confirmed co-infection results are consistent with the findings of all cases (Supplementary Table S6). The receiver operating characteristic curves in Figure 3 show that the combined use of CSF pressure and the concentrations of glucose, protein and chlorine in CSF might represent a promising discriminatory indicator (area under curve=0.89; Supplementary Table S7). However, the data were insufficient to determine cut-off values for the combined tests. Such values should be further investigated in larger studies.

### Treatment profile

The therapeutic strategies of most studies were not adequately described. In particular, information on the induction and consolidation therapy to treat cryptococcosis was lacking. Brief records of antifungal drug treatments were available for 148 cases, and 81 (54.7%) were considered to have received substandard treatments that did not strictly follow the intervention guidelines for cryptococcosis.^27^ More details are provided in Supplementary Table S8.

The outcomes of 149 cases were available, and the case fatality rate was 11.4%.

## DISCUSSION

TB/cryptococcosis co-infection is potentially an emerging problem in China, accounting for 62.9% of global cases. However, most cases were reported in Chinese and lacked an English language abstract, thereby hiding this striking trend and diagnostic challenge from non-Chinese researchers. Hence, we conducted a retrospective investigation to create an epidemiological and clinical summation, and performed a comparative analysis to identify useful laboratory indicators for differential diagnosis.

A total of 197 co-infection cases were collected since 1965 in China. A rapid increase in case numbers was observed, and more than half of these co-infections were reported after 2010. For prevalence studies, a large-scale study in Taiwan from 1993 to 2006 revealed that the rates of co-infection were 0.6% and 5.4% for TB and cryptococcosis, respectively. Furthermore, other studies reported prevalence data linked to conditions, such as TB meningitis (9.7%), TB/fungal co-infection (2.7%–2.9%), cryptococcosis in children (6.7%–26.7%) and cryptococcal meningitis with or without HIV/AIDS (42.9% and 15.0%, respectively). These data highlighted the likelihood of co-infection in specific high-risk populations. However, considering the small sample size of most of the included prevalence studies, additional large-scale multi-center studies are needed to confirm whether the changing trends in incidence and prevalence are a result of increased case numbers of TB, cryptococcosis and HIV or due to recently developed diagnostics. The notable nationwide increase in case numbers reflects the increasing morbidity of TB/cryptococcosis co-infection and the rising clinical awareness of this condition among clinicians in China.

Geographically, nearly all cases were clustered within the monsoon region, and the majority of cases were from southern and eastern China. Considering the epidemiological features of TB in China (Supplementary Figure S2), we speculated whether this distinct distribution of co-infection may be mainly caused by the environmental distribution of *Cryptococcus*. The monsoon region, particularly in southern and eastern China, is warmer and wetter than the rest of the country, and these conditions are known to facilitate the survival and reproduction of pathogens.^28^ Another explanation might be that this region is more economically and medically developed than other regions, which potentially leads to higher co-infection recognition rates because of improved diagnostics.

Despite the potential significance, the clinical profile of HIV–TB cryptococcosis triple infections remains poorly understood. Our study included a large number of patients with cryptococcosis living with both HIV/AIDS and TB infections. The TB and HIV/AIDS dual epidemic is currently an issue of international concern. TB is the leading killer among patients with HIV/AIDS, and 99% of this mortality occurs in resource-limited countries.^29^ Likewise, cryptococcosis is also a lethal complication among patients living with HIV/AIDS, generally contributing a fatality rate of 55% in less-developed regions.^16^ TB was also an independent risk factor for cryptococcosis, which could be explained by the innate immunity suppression it causes.^30^ The immunodeficiency caused by HIV is the greatest risk factor for patients with both TB and cryptococcosis, and their mutually detrimental effects aggravate the disease process. The current study described the clinical manifestations of triple infections in detail, and further comparisons suggested that patients with triple infections tended to correlate with more severe constitutional, respiratory and neurological symptoms than those with co-infections. However, the laboratory results of triple infections could not be used in a statistical analysis due to the small sample size of this group among our reviewed cases.

The cases of TB/cryptococcal co-infection in the present study were characterized by a high frequency of misdiagnoses according to the text of included studies. Although most of our cases were from tertiary hospitals, the misdiagnosis rate was 76.9%. The misdiagnosis rate reached an extremely high level (95.2%, *n*=40/42) for patients with tubercular/cryptococcal meningitis, which was the most damaging and most common co-infection. The definitive, early recognition of co-infection is the core difficulty for physicians, and this recognition is key to aggressive clinical intervention. However, non-specific presentations hamper the differentiation of poly-infections from mono-infections, particularly regarding brain infections. In addition, and contrary to the widespread use of TB diagnostic techniques, India ink staining and the latex agglutination test for *Cryptococcus* are not routine tests for patients with suspected brain infection in most Chinese hospitals. Consequently, incomplete diagnoses were responsible for 91.4% (*n*=64/70) of all misdiagnoses and 93.8% (*n*=60/64) of all cases of missed cryptococcosis. Therefore, given the high proportion of brain co-infection as well as the unavailability and unaffordability of extra advanced diagnostic tests for individuals living in developing nations, such as China, it is necessary to explore new indicators using basic laboratory tests that are easy to perform and impose no additional financial burden. Conclusive differentiation between tubercular/cryptococcal meningitis remains an underinvestigated but important topic. For the first time, our research revealed that the combined use of CSF pressure, glucose, protein and chlorine is a reliable discriminatory marker for CSF co-infections (AUC=0.89). This method helps to identify meningitis and suggests that further etiological examinations are required. CSF pressure and biochemical tests are routine for most patients with severe neurological symptoms, regardless of a suspected CSF infection. The processing speed (within 1 day) and low cost of CSF pressure, and biochemical tests make it a promising screening tool for tubercular meningitis, cryptococcal meningitis and tubercular/cryptococcal meningitis. In addition, other promising sensitive and specific targets, such as immunoglobulin and adenosine deaminase levels, may be useful adjunctive tests for the differential diagnosis of tubercular/cryptococcal meningitis, and these tests need to be evaluated in further studies.^31^

According to the guidelines of the Infectious Diseases Society of America, patients with cryptococcal meningitis, cryptococcemia or dissemination should take amphotericin B plus flucytosine, followed by fluconazole as a maintenance therapy. In addition, pulmonary cryptococcosis is treated with fluconazole.^27^ Unlike the continuous attention paid to TB control over several years, Chinese physicians continue to inadequately follow the standard therapy guidelines for the treatment of cryptococcosis. Our study revealed that more than half of all co-infection cases received substandard treatments for cryptococcosis, even though most were treated at tertiary hospitals in China. In addition, HIV–TB cryptococcosis triple infections might pose a new challenge to the dual global epidemics of HIV and TB. We recommend that additional efforts are made to explore new therapeutic strategies to treat triple infections, and further emphasis should be placed on creating an integrated therapy to control the HIV/TB dual pandemic to reduce potential complications due to poly-infection.

## Figures and Tables

**Table 1:** Clinical manifestations of TB/cryptococcosis co-infection with regard to HIV infection

**Presenting symptoms**	**Total**	**Patients without HIV**	**Patients with HIV**	***P*-value**
*Constitutional*				
Fever	83/110^a^ (41/54)^b^	59/80 (30/40)	24/30 (11/14)	0.50 (1.00)
Nausea	16/87 (6/31)	10/68 (4/28)	6/19 (2/3)	0.18 (0.12)
Vomiting	34/87 (13/31)	23/68 (11/28)	11/19 (2/3)	0.06 (0.77)
Weakness	30/87 (7/31)	15/68 (5/28)	15/19 (2/3)	0.00 (0.23)
Weight loss	14/110 (12/54)	5/80 (4/40)	9/19 (8/14)	0.00 (0.00)
Night sweats	14/110 (5/54)	7/80 (2/40)	7/30 (3/14)	0.10 (0.20)
Anorexia	10/110 (8/54)	6/80 (4/40)	4/30 (4/14)	0.57 (0.21)

*Respiratory*				
Cough	42/87 (5/31)	26/68 (3/28)	16/19 (2/3)	0 (0.09)
Sputum	37/87 (4/31)	22/68 (3/28)	15/19 (1/3)	0 (0.84)
Dyspnea	2/87 (1/31)	1/68 (0/28)	1/19 (1/3)	0.91 (0.17)
Chest pain	18/87 (1/31)	10/68 (1/28)	8/19 (0/3)	0.02 (1.00)

*Neurological*				
Headache	44/87 (22/31)	35/68 (19/28)	9/19 (3/3)	0.75 (0.62)
Signs of meningeal irritation	34/87 (15/31)	27/68 (14/28)	7/19 (1/3)	0.82 (1.00)
Conscious disturbance	14/87 (7/31)	11/68 (6/28)	3/19 (1/3)	1 (1.00)
Dizziness	3/87 (0/31)	1/68 (0/28)	2/19 (0/3)	0.23 (ND^c^)
Deep reflexes	4/87 (2/31)	3/68 (1/28)	1/19 (1/3)	1.00 (0.45)
Hearing loss	2/87 (2/31)	2/68 (2/28)	0/19 (0/3)	1.00 (1.00)
Vision disorders	4/87 (1/31)	4/68 (1/28)	0/19 (0/3)	0.64 (1.00)
Papilledema	10/87 (3/31)	4/68 (3/28)	6/19 (0/3)	0.01 (1.00)
Pupil reacted sluggishly to light	3/87 (0/31)	3/68 (0/28)	0/19 (0/3)	0.83 (ND)

Abbreviations: not determined, ND; tuberculosis, TB.

a Positive/total number of all cases.

b Positive/total number of cases etiologically diagnosed.

c No data.

**Table 2:** Univariate analysis comparing CSF variables among etiologically diagnosed patients with tubercular/cryptococcal meningitis, tubercular meningitis and cryptococcal meningitis

**CSF parameters**	**Tubercular/cryptococcal meningitis** **Mean (95% CI)**	**Tubercular meningitis** **Mean (95% CI)**	**Cryptococcal meningitis** **Mean (95% CI)**	***P*-value**
Intracranial pressure (mmH_2_O)	333.6 (266.9, 400.3) *n*=9	235.4 (212.0, 258.8) *n*=63	303.3 (284.2, 322.3) *n*=109	<0.05;^a^ >0.05;^b^ <0.05^c^
CSF glucose (mmol/L)	1.6 (1.1, 1.9) *n*=18	2.4 (1.9, 2.8) *n*=63	2.4 (2.2, 2.7) *n*=100	<0.05;^a^ <0.05;^b^ >0.05^c^
CSF protein (mg/L)	1620.2 (1035.6, 2204.8) *n*=16	1557.7 (1338.4, 1777.0) *n*=65	867.4 (711.6, 1023.2) *n*=104	>0.05;^a^ <0.05;^b^ <0.05^c^
CSF chloride (mmol/L)	114.6 (104.6, 124.5) *n*=17	115.1 (112.7, 117.5) *n*=58	121.5 (119.2, 123.8) *n*=92	>0.05;^a^ <0.05;^b^ <0.05^c^

Abbreviations: confidence interval, CI; cerebrospinal fluid, CSF.

a Tubercular/cryptococcal meningitis vs tubercular meningitis.

b Tubercular/cryptococcal meningitis vs cryptococcal meningitis.

c Tubercular meningitis vs cryptococcal meningitis.
