# Supplementary material for: Tuberculosis/cryptococcosis co-infection in China between 1965 and 2016
Source: Emerg Microbes Infect. 2017 Aug 23;6(8):e73–. doi: 10.1038/emi.2017.61 (PMC5583669; doi:10.1038/emi.2017.61)
Supplement: Supplementary Figure S1 [file emi201761x1.ppt]

## Slide 1
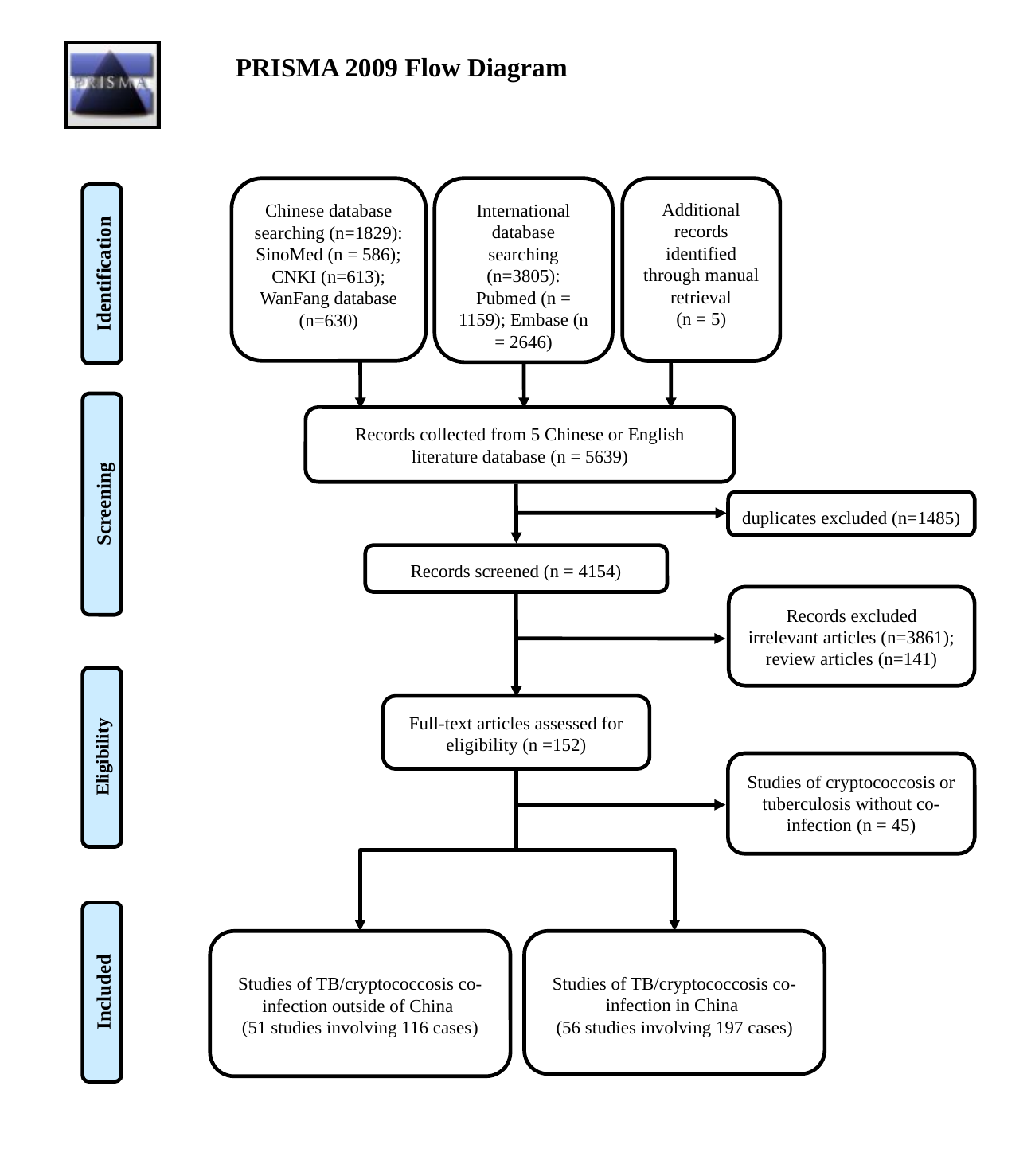

PRISMA 2009 Flow Diagram
Chinese database searching (n=1829): SinoMed (n = 586); CNKI (n=613); WanFang database (n=630)
International database searching (n=3805): Pubmed (n = 1159); Embase (n = 2646)
Additional records identified through manual retrieval
(n = 5)
Identification
Records collected from 5 Chinese or English literature database (n = 5639)
Screening
duplicates excluded (n=1485)
Records screened (n = 4154)
Records excluded
irrelevant articles (n=3861); review articles (n=141)
Full-text articles assessed for eligibility (n =152)
Eligibility
Studies of cryptococcosis or tuberculosis without co-infection (n = 45)
Studies of TB/cryptococcosis co-infection outside of China
(51 studies involving 116 cases)
Studies of TB/cryptococcosis co-infection in China
(56 studies involving 197 cases)
Included
