# Supplementary figures and images for: Tuberculosis/cryptococcosis co-infection in China between 1965 and 2016
Source: Emerg Microbes Infect. 2017 Aug 23;6(8):e73–. doi: 10.1038/emi.2017.61 (PMC5583669; doi:10.1038/emi.2017.61)

A

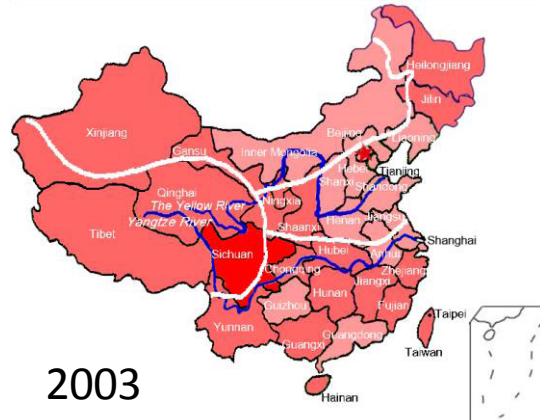

B

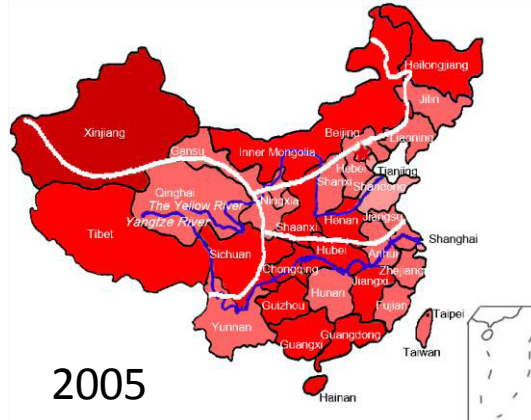

C

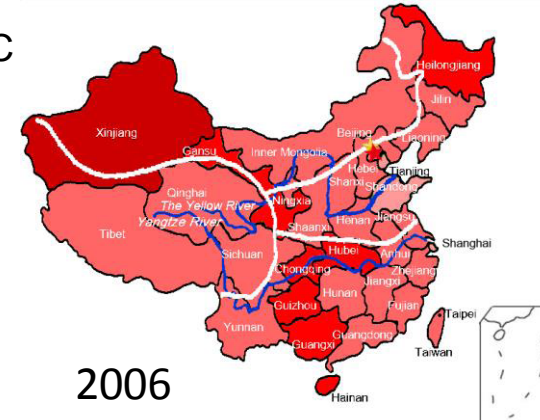

D

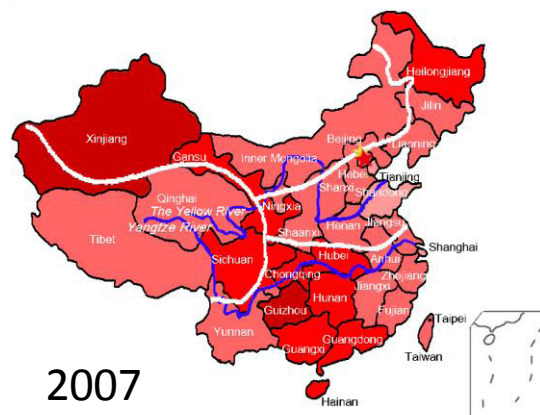

E

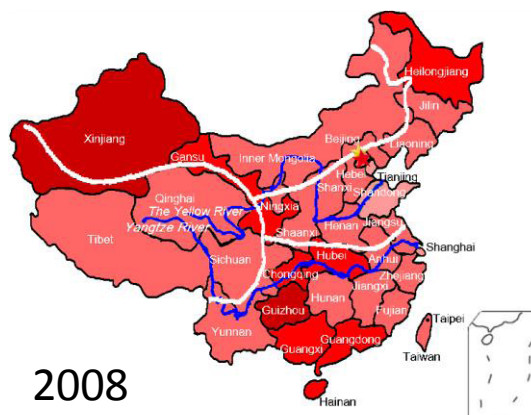

F

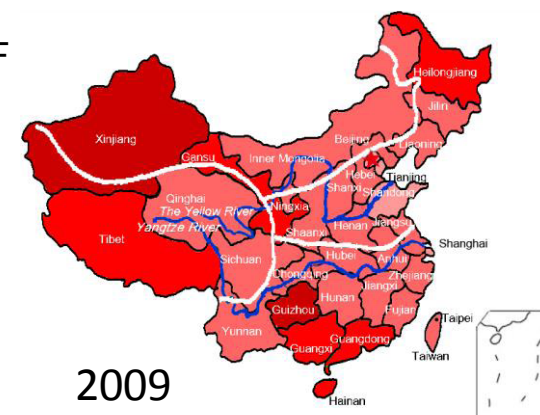

G

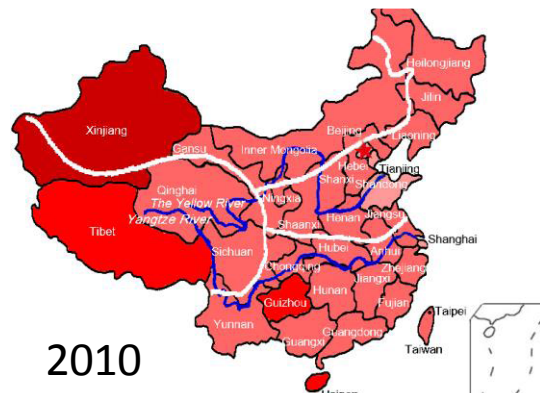

H

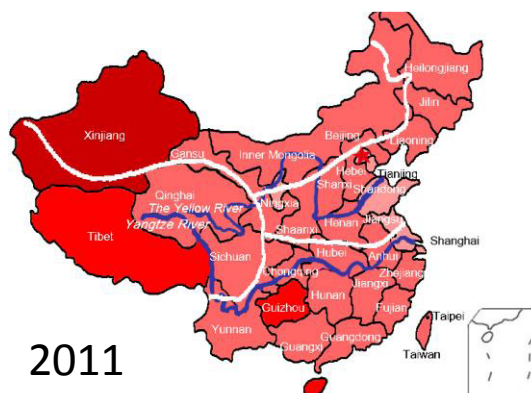

I

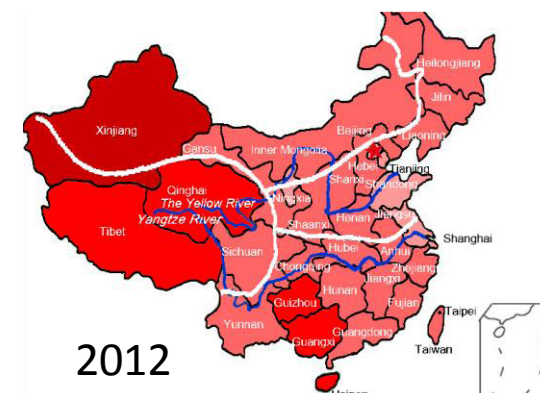

>150 100-150 50-100 <50 Incidence of tuberculosis in China (100000 population per year)

Supplement: Supplementary Figure S2 [file emi201761x2.pdf]
