# Supplementary material for: Tuberculosis/cryptococcosis co-infection in China between 1965 and 2016
Source: Emerg Microbes Infect. 2017 Aug 23;6(8):e73–. doi: 10.1038/emi.2017.61 (PMC5583669; doi:10.1038/emi.2017.61)
Supplement: Supplementary Table S1 [file emi201761x3.docx]

**Supplementary Table S1.** Epidemiology characteristics of patients with TB/cryptococcosis co-infection

| Study number | DOI/websites^a^ | Publication year | Number of cases | Sex^d^ | Age | Affected site of the co-infection | Underlying diseases | Geographical location | Prognosis |
| --- | --- | --- | --- | --- | --- | --- | --- | --- | --- |
| 1 | http://www.cnki.com.cn/Article/CJFDTotal-ZFLZ196503035.htm | 1965 | 1 | F | 31 | Cryptococcosis (Brain) + TB (Lung) | No | Beijing | ND |
| 2 | http://www.cnki.com.cn/Article/CJFDTotal-ZSEK198904008.htm | 1989 | 1^b^ | M | 2 | Cryptococcosis (Brain) + TB (Brain+Lung) | No | Mudanjiang, Heilongjiang | Died |
| 3 | http://www.cqvip.com/qk/92358X/199104/676570.html | 1991 | 1 | M | 40 | Cryptococcosis (Brain) + TB (Brain) | No | Shanghai | Survived |
|  |  |  | 1 | M | 48 | Cryptococcosis (Brain) + TB (Brain) | No | Shanghai | Survived |
|  |  |  | 1^b^ | M | 39 | Cryptococcosis (Brain) + TB (Brain) | No | Shanghai | Survived |
| 4 | http://www.cnki.com.cn/Article/CJFDTotal-ZSEK199403028.htm | 1994 | 1^b^ | F | 3 | Cryptococcosis (Brain) + TB (Brain+Lung) | No | Suihua, Heilongjiang | Survived |
| 5 | http://www.cnki.com.cn/Article/CJFDTotal-TJYK603.044.htm | 1996 | 1 | M | 43 | Cryptococcosis (Brain) + TB (Brain) | No | Mudanjiang, Heilongjiang | Died |
| 6 | http://www.cnki.com.cn/Article/CJFDTotal-JLYX701.053.htm | 1997 | 1 | M | 24 | Cryptococcosis (Brain) + TB (Brain+Lung) | No | Changchun, Jilin | Survived |
| 7 | http://www.cnki.com.cn/Article/CJFDTotal-XDJH199811138.htm | 1998 | 1^b^ | M | 2 | Cryptococcosis (Brain) + TB (Brain) | No | Huaian, Jiangsu | Died |
| 8 | http://www.cnki.com.cn/Article/CJFDTotal-GZYY805.060.htm | 1998 | 1^b^ | M | 21 | Cryptococcosis (Brain) + TB (Brain) | No | Guangzhou, Guangdong | Died |
|  |  |  | 1^b^ | M | 33 | Cryptococcosis (Brain) + TB (Brain) | No | Guangzhou, Guangdong | Died |
| 9 | http://www.cnki.com.cn/Article/CJFDTotal-LYSJ199801033.htm | 1998 | 3 | ND | ND | Cryptococcosis (Brain) + TB (Lung) | ND | Shijiazhuang, Hebei | ND |
| 10 | http://www.cqvip.com/Main/Detail.aspx?id=3869124 | 1999 | 1^b^ | F | 25 | Cryptococcosis (Brain) + TB (Brain) | Subacute thyroiditis | Baotou, Inner Mongolia | Survived |
| 11 | <http://www.cnki.com.cn/Article/CJFDTotal-YXLL199904024.htm> | 1999 | 1 | F | 52 | Cryptococcosis (Brain) + TB (Lung) | No | Huian, Fujian | Died |
| 12 | 10.3321/j.issn:1009-7708.2001.01.013 | 2001 | 6 | ND | ND | Cryptococcosis (Brain) + TB (Lung) | No | Zhanjiang, Guangdong | ND |
| 13 | 10.3969/j.issn.1004-0501.2001.03.041 | 2001 | 1^b^ | ND | ND | Cryptococcosis (Brain) + TB (Brain) | No | Dazhou, Sichuan | ND |
| 14 | 10.3969/j.issn.1004-583X.2002.04.038 | 2002 | 1 | M | 53 | Cryptococcosis (Brain) + TB (Lung and bone) | No | Changsha, Hunan | Survived |
|  |  |  | 1 | F | 48 | Cryptococcosis (Brain) + TB (Brain) | No | Changsha, Hunan | Survived |
| 15 | 10.3969/j.issn.1002-266X.2003.07.024 | 2003 | 1 | M | 47 | Cryptococcosis (Brain) + TB (Brain) | No | Jinan, Shandong | Survived |
| 16 | 10.3760/cma.j.issn.1672-7088.2003.15.040 | 2003 | 1^b^ | F | 4 | Cryptococcosis (Brain) + TB (Brain+Lung) | No | Chongqing | Survived |
| 17 | 10.3969/j.issn.1000-2294.2003.04.051 | 2003 | 1 | M | 42 | Cryptococcosis (Blood) + TB (Liver) | No | Yudu, Jiangxi | Survived |
| 18 | 10.3969/j.issn.1004-583X.2003.17.033 | 2003 | 3 | ND | ND | Cryptococcosis (Brain) + TB (Lung) | ND | Luoyang, Henan | ND |
| 19 | 10.3969/j.issn.1672-6731.2004.01.019 | 2004 | 1^b^ | M | 18 | Cryptococcosis (Brain) + TB (Lung) | No | Chuxiong, Yunnan | Survived |
| 20 | 10.3969/j.issn.1000-3711.2005.02.055 | 2005 | 1 | M | 27 | Cryptococcosis (Brain) + TB (Brain) | HIV; Syphilis | Urumqi, Xinjiang | Died |
| 21 | http://www.doc88.com/p-4724413433780.html | 2005 | 1^b^ | M | 16 | Cryptococcosis (Brain) + TB (Brain+Lung) | No | Panjin, Liaoning | Survived |
| 22 | 10.3969/j.issn.1009-8194.2005.10.034 | 2005 | 1^b^ | M | 48 | Cryptococcosis (Brain) + TB (Brain) | No | Nanchang, Jiangxi | Survived |
|  |  |  | 1^b^ | M | 62 | Cryptococcosis (Brain) + TB (Brain+Lung) | No | Nanchang, Jiangxi | Died |
|  |  |  | 1^b^ | M | 30 | Cryptococcosis (Brain) + TB (Brain+Lung) | No | Nanchang, Jiangxi | Survived |
| 23 | 10.3969/j.issn.1008-8849.2005.15.077 | 2005 | 1 | F | 21 | Cryptococcosis (Brain) + TB (Brain) | No | Panjin, Liaoning | Survived |
| 24 | 10.1038/emi.2016.95 | 2005 | 1^b^ | M | 31 | Cryptococcosis (Brain) + TB (Brain) | No | Shanghai | Survived |
| 25 | 10.3969/j.issn.1000-6621.2005.06.008 | 2005 | 2^b^ | ND | ND | Cryptococcosis (Lung) + TB (Lung) | ND | Jinan, Shandong | ND |
| 26 | 10.3969/j.issn.1671-8348.2005.02.008 | 2005 | 4^c^ | ND | ND | Cryptococcosis (Lung) + TB (Lung) (N=2)；Cryptococcosis (Brain) + TB (Lung)；Cryptococcosis (Brain) + TB (disseminated) | ND | Chongqing | ND |
| 27 | http://www.cnki.com.cn/Article/CJFDTotal-ZFLZ2006S1057.htm | 2006 | 1^b^ | M | 47 | Cryptococcosis (Brain) + TB (Brain) | No | Chongqing | Survived |
| 28 | 10.3969/j.issn.1000-6621.2006.05.019 | 2006 | 13 | M (n=9); F (n=4) | 47.7 [18, 65] | Cryptococcosis (Lung) + TB (Lung) | No | Liuzhou, Guangxi | ND |
| 29 | 10.3760/j:issn:0376-2491.2007.27.017 | 2007 | 1^b^ | M | 56 | Cryptococcosis (Blood+Skin) + TB (Blood) | Diabetes | Beijing | Died |
| 30 | 10.3969/j.issn.1009-6647.2007.24.100 | 2007 | 3 | M (n=3) | [19, 46] | Cryptococcosis (Brain) + TB (Brain) | No | Anshan, Liaoning | Survived |
| 31 | 10.3969/j.issn.1000-7377.2007.11.044 | 2007 | 5 | M (n=5) | [19, 62] | Cryptococcosis (Brain) + TB (Brain) | No | Jinggangshan, Jiangxi | Died (n=1); Survived (n=4) |
| 32 | 10.3969/j.issn.1009-0959.2012.03.059 | 2009 | 1^b^ | M | 65 | Cryptococcosis (Lung+Skin)+ TB (Lung) | No | Beijing | Survived |
| 33 | 10.3969/j.issn.1671-6450.2009.09.007 | 2009 | 1^b^ | M | 39 | Cryptococcosis (Brain) + TB (Brain) | HIV | Nanning, Guangxi | Survived |
| 34 | 10.3760/cma.j.issn.1674-2397.2009.05.017 | 2009 | 1^b^ | M | 21 | Cryptococcosis (Brain) + TB (Brain +Lung) | No | Wenzhou, Zhejiang | Survived |
|  |  |  | 1^b^ | M | 30 | Cryptococcosis (Brain) + TB (Brain +Lung) | No | Wenzhou, Zhejiang | Died |
| 35 | http://d.wanfangdata.com.cn/Periodical/zgflzz200909018 | 2009 | 8 | M (n=5); F (n=3) | 42 [23, 63] | Cryptococcosis (Lung) + TB (Lung) | SLE (n=1); Diabetes (n=1) | Shanghai | Survived (n=8) |
| 36 | 10.3969/j.issn.1007-614x.2009.07.052 | 2009 | 6 | ND | ND | Cryptococcosis (Brain) + TB (Lung) | HIV (n=6) | Puer, Yunnan | ND |
| 37 | http://d.wanfangdata.com.cn/Periodical/zgwzxzz201018233 | 2010 | 1 | M | 22 | Cryptococcosis (Brain) + TB (Brain) | No | Shiyan, Hubei | Survived |
| 38 | 10.3969/j.issn.0253-4304.2010.09.066 | 2010 | 11^b^ | M (n=5); F (n=6) | 36.8 [18, 70] | Cryptococcosis (Lung) + TB (Lung) | HIV (n=11) | Yulin, Guangxi | Survived |
| 39 | 10.1007/s15010-010-0045- | 2010 | 23^b^ | M (n=17); F (n=6) | 52±15 [25, 78] | ND | Diabetes (n=3); Liver cirrhosis (n=2); Alcoholism (n=2); Malignancy (n=2); Chronic kidney disease (n=1) | Taipei, Taiwan | Died (n=3) survived (n=20) |
| 40 | 10.3969/j.issn.1001-9448.2011.07.006 | 2011 | 1^b^ | M | 22 | Cryptococcosis (Brain) + TB (Brain) | No | Fuzhou, Fujian | Survived |
| 41 | 10.3760/cma.j.issn.1671-7368.2011.07.034 | 2011 | 1^b^ | F | 32 | Cryptococcosis (Brain) + TB (Lung) | SLE | Beijing | Survived |
| 42 | http://d.wanfangdata.com.cn/Periodical/cqykdxxb201108042 | 2011 | 1^b^ | M | 74 | Cryptococcosis (Brain) + TB (Lung) | HIV | Chengdu, Sichuan | Died |
| 43 | http://www.cnki.com.cn/Article/CJFDTotal-ZGYA201136031.htm | 2011 | 52 | M (n=38);  F (n=14) | 48.5±2.7 [22, 76] | Cryptococcosis (Brain) + TB (Brain) | ND | Nanning, Guangxi | Survived |
| 44 | 10.3969/j.issn.1006-5725.2011.23.037 | 2011 | 1^b^ | ND | ND | Cryptococcosis (Lung) + TB (Lung) | ND | Guangzhou, Guangdong | ND |
| 45 | 10.3969/j.issn.1672-5085.2011.05.256 | 2011 | 1 | F | 26 | Cryptococcosis (Brain) + TB (Brain) | No | Baotou, Inner Mongolia | survived |
| 46 | 10.3877/cma.j.issn.1674-0785.2012.24.096 | 2012 | 1 | M | 74 | Cryptococcosis (adrenal gland) + TB (Lung) | Diabetes | Beijing | Survived |
| 47 | http://d.wanfangdata.com.cn/Periodical/zjyx201222025 | 2012 | 1^b^ | M | 47 | Cryptococcosis (Lung) + TB (Lung) | No | Hangzhou, Zhejiang | Survived |
| 48 | http://d.wanfangdata.com.cn/Periodical/zgwzxzz201217177 | 2012 | 1^b^ | ND | ND | Cryptococcosis (Lung) + TB (Lung) | ND | Zhengzhou, Henan | ND |
| 49 | http://d.wanfangdata.com.cn/Periodical/zgfybj201231028 | 2012 | 1 | ND | ND | Cryptococcosis (Lung) + TB (Lung) | ND | Zhengzhou, Henan | ND |
| 50 | 10.3969/j.issn.1671-6450.2013.10.030 | 2013 | 1^b^ | M | 48 | Cryptococcosis (Brain) + TB (Lung) | HIV; Hepatitis B | Baoding, Hebei | Died |
| 51 | http://d.wanfangdata.com.cn/Periodical/zgyyyypjyfx201309031 | 2013 | 1^b^ | M | 16 | Cryptococcosis (Lung) + TB (Lung) | No | Guangzhou, Guangdong | Survived |
| 52 | 10.3969/j.issn.1004-0501.2013.07.027 | 2013 | 5 | M (n=3); F (n=2) | 49.6±7.02 [17, 76] | Cryptococcosis (Brain) + TB (Brain) | Diabetes (n=3); HIV (n=1); Renal insufficiency (n=1) | Chengdu, Sichuan | Died (n=1); Survived (n=4) |
| 53 | 10.1093/qjmed/hct211 | 2014 | 1^b^ | F | 58 | Cryptococcosis (Lung) + TB (neck lymph node) | No | Taipei, Taiwan | Survived |
| 54 | 10.3969/j.issn.1006-1959.2015.45.272 | 2015 | 4 | M (n=4) | [37, 58] | Cryptococcosis (Brain) + TB (Lung) | HIV (n=4); hepatitis B (n=1); Hepatitis C (n=1); syphilis (n=2) | Jingzhou, Hubei | ND |
| 55 | 10.3969/j.issn.1000-0952.2015.19.049 | 2015 | 1 | M | 17 | Cryptococcosis (Lung) + TB (Lung) | No | Dingxi, Gansu | Survived |
| 56 | 10.14163/j.cnki.11-5547/r.2015.20.055 | 2015 | 2 | ND | ND | Cryptococcosis (Lung) + TB (Lung) | No | Jinzhou, Liaoning | ND |

Note: a: Chinese DOI: <http://www.chinadoi.cn/portal/index.htm>; b: TB/cryptococcosis co-infection was etiologically diagnosed; c: co-infection of 2 cases were etiologic diagnosed; d: M: male; F: female; ND: no data;
