# Supplementary material for: Tuberculosis/cryptococcosis co-infection in China between 1965 and 2016
Source: Emerg Microbes Infect. 2017 Aug 23;6(8):e73–. doi: 10.1038/emi.2017.61 (PMC5583669; doi:10.1038/emi.2017.61)
Supplement: Supplementary Table S2 [file emi201761x4.docx]

**Supplementary Table S2**. Clinical manifestations of TB/cryptococcosis co-infection

| Study number | Constitutional signs | | | | | | | Respiratory signs | | | | | | Neurological signs | | | | | | | | |
| --- | --- | --- | --- | --- | --- | --- | --- | --- | --- | --- | --- | --- | --- | --- | --- | --- | --- | --- | --- | --- | --- | --- |
|  | Fever | Nausea | Vomiting | Weakness | Weight loss | Night sweats | Anorexia |  | Cough | Sputum | Dyspnea | Chest pain |  | Headache | Signs of meningeal  irritation | Conscious disturbance | Dizziness | Deep reflexes | Hearing loss | Vision  disorders | Papilledema | Pupil reacted sluggishly to light |
| 1 | Pos (n=1) | Neg | Pos (n=1) | Neg | Neg | Neg | Neg |  | Neg | Neg | Neg | Neg |  | Pos (n=1) | Neg | Neg | Neg | Neg | Neg | Neg | Neg | Neg |
| 2 | Pos | Neg | Pos | Pos | Pos | Neg | Pos |  | Neg | Neg | Neg | Neg |  | Neg | Neg | Neg | Neg | Neg | Neg | Neg | Neg | Neg |
| 3 | Pos | Neg | Pos | Neg | Neg | Neg | Neg |  | Neg | Neg | Neg | Neg |  | Pos | Pos | Neg | Neg | Neg | Neg | Neg | Neg | Neg |
|  | Pos | Neg | Pos | Pos | Neg | Neg | Neg |  | Neg | Neg | Neg | Neg |  | Pos | Pos | Neg | Neg | Neg | Neg | Neg | Neg | Neg |
|  | Pos | Neg | Pos | Pos | Neg | Neg | Neg |  | Neg | Neg | Neg | Neg |  | Pos | Pos | Neg | Neg | Neg | Neg | Neg | Neg | Neg |
| 4 | Pos | Pos | Pos | Pos | Pos | Pos | Pos |  | Neg | Neg | Neg | Neg |  | Pos | Pos | Neg | Neg | Pos | Neg | Neg | Neg | Neg |
| 5 | Pos | Neg | Pos | Neg | Neg | Neg | Neg |  | Neg | Neg | Neg | Neg |  | Pos | Pos | Pos | Neg | Neg | Neg | Neg | Neg | Pos |
| 6 | Pos | Neg | Pos | Pos | Pos | Neg | Neg |  | Pos | Neg | Neg | Neg |  | Pos | Pos | Neg | Neg | Neg | Neg | Neg | Neg | Neg |
| 7 | Neg | Neg | Pos | Pos | Neg | Neg | Neg |  | Neg | Neg | Neg | Neg |  | Neg | Pos | Neg | Neg | Neg | Neg | Neg | Neg | Neg |
| 8 | Neg | Neg | Pos | Neg | Neg | Neg | Neg |  | Neg | Neg | Neg | Neg |  | Neg | Pos | Neg | Neg | Neg | Neg | Neg | Neg | Neg |
|  | Pos | Neg | Pos | Neg | Neg | Neg | Neg |  | Neg | Neg | Neg | Neg |  | Pos | Pos | Neg | Neg | Neg | Neg | Neg | Neg | Neg |
| 9 | ND | ND | ND | ND | ND | ND | ND |  | ND | ND | ND | ND |  | ND | ND | ND | ND | ND | ND | ND | ND | ND |
| 10 | Pos | Neg | Neg | Neg | Neg | Neg | Neg |  | Neg | Neg | Neg | Neg |  | Pos | Pos | Neg | Neg | Neg | Neg | Neg | Neg | Neg |
| 11 | Neg | Neg | Neg | Neg | Neg | Neg | Neg |  | Pos | Pos | Neg | Neg |  | Pos | Pos | Neg | Neg | Neg | Neg | Pos | Neg | Neg |
| 12 | ND | ND | ND | ND | ND | ND | ND |  | ND | ND | ND | ND |  | ND | ND | ND | ND | ND | ND | ND | ND | ND |
| 13 | Pos (n=5) | Neg | Neg | Neg | Neg | Neg | Neg |  | Neg | Neg | Neg | Neg |  | Pos (n=5) | Neg | Pos (n=4) | Neg | Neg | Neg | Neg | Neg | Neg |
| 13 | ND | ND | ND | ND | ND | ND | ND |  | ND | ND | ND | ND |  | ND | ND | ND | ND | ND | ND | ND | ND | ND |
| 14 | Pos | Neg | Pos | Neg | Neg | Neg | Neg |  | Neg | Neg | Neg | Neg |  | Pos | Pos | Neg | Neg | Pos | Neg | Neg | Neg | Neg |
|  | Pos | Pos | Pos | Neg | Neg | Neg | Neg |  | Neg | Neg | Neg | Neg |  | Neg | Pos | Neg | Neg | Neg | Neg | Pos | Neg | Pos |
| 15 | Pos | Neg | Neg | Neg | Neg | Neg | Neg |  | Neg | Neg | Neg | Neg |  | Pos | Pos | Neg | Neg | Neg | Neg | Neg | Neg | Neg |
| 16 | Pos | Neg | Neg | Pos | Neg | Neg | Neg |  | Neg | Neg | Neg | Neg |  | Neg | Neg | Neg | Neg | Neg | Neg | Neg | Neg | Neg |
| 17 | Pos | Pos | Pos | Neg | Neg | Neg | Pos |  | Pos | Neg | Neg | Neg |  | Pos | Pos | Neg | Neg | Neg | Neg | Neg | Neg | Neg |
| 18 | ND | ND | ND | ND | ND | ND | ND |  | ND | ND | ND | ND |  | ND | ND | ND | ND | ND | ND | ND | ND | ND |
| 19 | Pos | Neg | Pos | Neg | Neg | Neg | Neg |  | Neg | Neg | Neg | Neg |  | Pos | Pos | Neg | Neg | Neg | Neg | Neg | Pos | Neg |
| 20 | Pos | Pos | Pos | Pos | Pos | Pos | Neg |  | Pos | Pos | Neg | Neg |  | Neg | Neg | Neg | Neg | Neg | Neg | Neg | Neg | Neg |
| 21 | Pos | Neg | Neg | Neg | Neg | Neg | Neg |  | Neg | Neg | Neg | Neg |  | Pos | Pos | Neg | Neg | Neg | Neg | Neg | Pos | Neg |
| 22 | Pos | Neg | Neg | Neg | Neg | Neg | Neg |  | Neg | Neg | Neg | Neg |  | Pos | Pos | Neg | Neg | Neg | Neg | Neg | Neg | Neg |
|  | Pos | Neg | Neg | Neg | Neg | Neg | Neg |  | Neg | Neg | Neg | Neg |  | Pos | Pos | Neg | Neg | Neg | Neg | Neg | Neg | Neg |
|  | Pos | Neg | Neg | Neg | Neg | Neg | Neg |  | Neg | Neg | Neg | Neg |  | Pos | Pos | Neg | Neg | Neg | Neg | Neg | Neg | Neg |
| 23 | Pos | Neg | Neg | Neg | Neg | Neg | Neg |  | Neg | Neg | Neg | Neg |  | Pos | Pos | Pos | Neg | Neg | Neg | Pos | Pos | Pos |
| 24 | Pos | Pos | Pos | Neg | Neg | Neg | Neg |  | Neg | Neg | Neg | Neg |  | Pos | Neg | Neg | Neg | Neg | Neg | Neg | Neg | Neg |
| 25 | ND | ND | ND | ND | ND | ND | ND |  | ND | ND | ND | ND |  | ND | ND | ND | ND | ND | ND | ND | ND | ND |
| 26 | ND | ND | ND | ND | ND | ND | ND |  | ND | ND | ND | ND |  | ND | ND | ND | ND | ND | ND | ND | ND | ND |
| 27 | Pos | Neg | Pos | Neg | Neg | Neg | Neg |  | Neg | Neg | Neg | Neg |  | Pos | Pos | Pos | Neg | Neg | Pos | Neg | Neg | Neg |
| 28 | Pos (n=6) | Neg | Neg | Pos (n=6) | Neg | Neg | Neg |  | Pos (n=13) | Pos (n=13) | Neg | Pos (n=5) |  | Neg | Neg | Neg | Neg | Neg | Neg | Neg | Neg | Neg |
| 29 | Pos | Neg | Neg | Neg | Neg | Neg | Neg |  | Neg | Neg | Neg | Neg |  | Neg | Neg | Neg | Neg | Neg | Neg | Neg | Neg | Neg |
| 30 | Pos (n=3) | Pos (n=3) | Pos (n=3) | Neg | Neg | Neg | Neg |  | Neg | Neg | Neg | Neg |  | Pos (n=3) | Neg | Pos (n=2) | Neg | Neg | Neg | Neg | Neg | Neg |
| 32 | Pos | Neg | Neg | Neg | Neg | Neg | Neg |  | Pos | Pos | Neg | Neg |  | Neg | Neg | Neg | Neg | Neg | Neg | Neg | Neg | Neg |
| 33 | Pos | Pos | Pos | Pos | Pos | Pos | Neg |  | Neg | Neg | Neg | Neg |  | Pos | Pos | Neg | Neg | Pos | Neg | Neg | Neg | Neg |
| 34 | Pos | Pos | Pos | Neg | Neg | Neg | Neg |  | Neg | Neg | Neg | Neg |  | Pos | Pos | Neg | Neg | Neg | Neg | Neg | Neg | Neg |
|  | Pos | Neg | Neg | Neg | Neg | Neg | Neg |  | Pos | Pos | Neg | Neg |  | Pos | Pos | Pos | Neg | Neg | Pos | Neg | Neg | Neg |
| 35 | Pos (n=5) | Neg | Neg | Neg | Neg | Pos (n=5) | Neg |  | Pos (n=5) | Pos (n=3) | Neg | Pos (n=3) |  | Neg | Neg | Neg | Neg | Neg | Neg | Neg | Neg | Neg |
| 36 | ND | ND | ND | ND | ND | ND | ND |  | ND | ND | ND | ND |  | ND | ND | ND | ND | ND | ND | ND | ND | ND |
| 37 | Pos | Neg | Neg | Pos | Neg | Neg | Neg |  | Neg | Neg | Neg | Neg |  | Pos | Pos | Neg | Neg | Pos | Neg | Neg | Neg | Neg |
| 38 | Pos (n=9) | Neg | Pos (n=5) | Pos (n=9) | Neg | Neg | Neg |  | Pos (n=10) | Pos (n=10) | Neg | Pos (n=8) |  | Pos (n=3) | Pos (n=5) | Neg | Pos (n=2) | Neg | Neg | Neg | Pos (n=6) | Neg |
| 39 | Pos (n=13) | Neg | Neg | Neg | Pos (n=8) | Pos (n=3) | Pos (n=5) |  | Neg | Neg | Neg | Neg |  | Neg | Neg | Neg | Neg | Neg | Neg | Neg | Neg | Neg |
| 40 | Pos | Pos | Pos | Neg | Neg | Neg | Neg |  | Neg | Neg | Neg | Neg |  | Pos | Pos | Neg | Neg | Neg | Neg | Neg | Neg | Neg |
| 41 | Pos | Pos | Pos | Neg | Neg | Neg | Neg |  | Neg | Neg | Neg | Neg |  | Pos | Neg | Neg | Neg | Neg | Neg | Pos | Pos | Neg |
| 42 | Pos | Neg | Neg | Neg | Neg | Neg | Neg |  | Pos | Neg | Pos | Neg |  | Pos | Neg | Neg | Neg | Neg | Neg | Neg | Neg | Neg |
| 43 | ND | ND | ND | ND | ND | ND | ND |  | ND | ND | ND | ND |  | ND | ND | ND | ND | ND | ND | ND | ND | ND |
| 44 | ND | ND | ND | ND | ND | ND | ND |  | ND | ND | ND | ND |  | ND | ND | ND | ND | ND | ND | ND | ND | ND |
| 45 | Pos (n=1) | Neg | Neg | Neg | Neg | Neg | Neg |  | Pos (n=1) | Pos (n=1) | Neg | Neg |  | Pos (n=1) | Pos (n=1) | Pos (n=1) | Neg | Neg | Neg | Neg | Neg | Neg |
| 46 | Pos | Neg | Neg | Pos | Neg | Neg | Pos |  | Neg | Neg | Neg | Neg |  | Neg | Neg | Neg | Neg | Neg | Neg | Neg | Neg | Neg |
| 47 | Neg | Neg | Neg | Neg | Neg | Neg | Neg |  | Neg | Neg | Neg | Neg |  | Neg | Neg | Neg | Neg | Neg | Neg | Neg | Neg | Neg |
| 48 | ND | ND | ND | ND | ND | ND | ND |  | ND | ND | ND | ND |  | ND | ND | ND | ND | ND | ND | ND | ND | ND |
| 49 | ND | ND | ND | ND | ND | ND | ND |  | ND | ND | ND | ND |  | ND | ND | ND | ND | ND | ND | ND | ND | ND |
| 50 | Pos | Pos | Pos | Pos | Pos | Neg | Pos |  | Pos | Pos | Neg | Neg |  | Pos | Neg | Pos | Neg | Neg | Neg | Neg | Neg | Neg |
| 51 | Pos | Neg | Neg | Neg | Neg | Neg | Neg |  | Neg | Neg | Neg | Pos |  | Neg | Neg | Neg | Neg | Neg | Neg | Neg | Neg | Neg |
| 52 | Pos (n=5) | Pos (n=3) | Pos (n=3) | Neg | Neg | Neg | Neg |  | Neg | Neg | Neg | Neg |  | Pos (n=5) | Pos (n=4) | Pos (n=2) | Neg | Pos (n=1) | Neg | Pos (n=1) | Neg | Neg |
| 53 | Pos (n=1) | Neg | Neg | Neg | Neg | Neg | Neg |  | Pos (n=1) | Pos (n=1) | Neg | Neg |  | Neg | Neg | Neg | Neg | Neg | Neg | Neg | Neg | Neg |
| 54 | Pos (n=3) | Pos (n=3) | Pos (n=3) | Pos (n=3) | Neg | Pos (n=3) | Neg |  | Pos (n=3) | Pos (n=3) | Neg | Neg |  | Pos (n=3) | Pos (n=1) | Pos (n=2) | Neg | Neg | Neg | Neg | Neg | Neg |
| 55 | Pos (n=1) | Neg | Neg | Neg | Neg | Neg | Neg |  | Pos (n=1) | Pos (n=1) | Pos (n=1) | Pos (n=1) |  | Neg | Neg | Neg | Pos (n=1) | Neg | Neg | Neg | Neg | Neg |
| 56 | ND | ND | ND | ND | ND | ND | ND |  | ND | ND | ND | ND |  | ND | ND | ND | ND | ND | ND | ND | ND | ND |
