# Supplementary material for: Tuberculosis/cryptococcosis co-infection in China between 1965 and 2016
Source: Emerg Microbes Infect. 2017 Aug 23;6(8):e73–. doi: 10.1038/emi.2017.61 (PMC5583669; doi:10.1038/emi.2017.61)
Supplement: Supplementary Table S3 [file emi201761x5.docx]

**Supplementary Table S3**. The CSF variables of etiologically diagnosed tubercular/cryptococcal meningitis

| Study number | Affected site of the co-infection | Appearance | Intracranial pressure (mmH2O) | Glucose (mmol/L) | Protein (mg/L) | Chlorine (mmol/L) |
| --- | --- | --- | --- | --- | --- | --- |
| 2 | Cryptococcosis (Brain) + TB (Brain+Lung) | Turbid/yellow | ND | 0.56 | 3000 | 105.4 |
| 3 | Cryptococcosis (Brain) + TB (Brain) | Turbid/yellow | ND | 1.22 | 195 | 146.9 |
| 4 | Cryptococcosis (Brain) + TB (Brain+Lung) | Turbid | 197 | 2.80 | ND | 103.0 |
| 7 | Cryptococcosis (Brain) + TB (Brain) | Turbid | ND | 2.00 | 1700 | 126.0 |
| 8 | Cryptococcosis (Brain) + TB (Brain) | Clear/colorless | ND | 0.51 | 2130 | 82.0 |
| 8 | Cryptococcosis (Brain) + TB (Brain) | Clear/colorless | ND | 2.80 | 4224 | 96.0 |
| 10 | Cryptococcosis (Brain) + TB (Brain) | Clear/colorless | 95 | 0.15 | 5400 | 86.0 |
| 19 | Cryptococcosis (Brain) + TB (Brain) | Clear/colorless | 400 | 0.54 | ND | ND |
| 21 | Cryptococcosis (Brain) + TB (Brain) | Clear/colorless | 350 | 2.40 | 3450 | 107.0 |
| 22 | Cryptococcosis (Brain) + TB (Brain+Lung) | Clear/colorless | ND | 1.10 | 1850 | 108.0 |
| 22 | Cryptococcosis (Brain) + TB (Brain+Lung) | Clear/colorless | ND | 0.90 | 1300 | 112.0 |
| 22 | Cryptococcosis (Brain) + TB (Brain) | Clear/colorless | ND | 0.60 | 2200 | 115.0 |
| 24 | Cryptococcosis (Brain) + TB (Brain) | Clear/colorless | 400 | 2.78 | 119 | 123.0 |
| 27 | Cryptococcosis (Brain) + TB (Brain) | Clear/colorless | 360 | 2.70 | 1680 | 103.0 |
| 33 | Cryptococcosis (Brain) + TB (Brain) | Turbid/yellow | ND | 2.96 | 1245 | 98.6 |
| 34 | Cryptococcosis (Brain) + TB (Brain +Lung) | Clear/colorless | 450 | 0.10 | 600 | 118.0 |
| 34 | Cryptococcosis (Brain) + TB (Brain +Lung) | Clear/colorless | 450 | 1.00 | 1100 | 117.0 |
| 40 | Cryptococcosis (Brain) + TB (Brain) | Clear/colorless | 300 | 1.90 | 1450 | 103.0 |
