# Supplementary material for: Tuberculosis/cryptococcosis co-infection in China between 1965 and 2016
Source: Emerg Microbes Infect. 2017 Aug 23;6(8):e73–. doi: 10.1038/emi.2017.61 (PMC5583669; doi:10.1038/emi.2017.61)
Supplement: Supplementary Table S4 [file emi201761x6.docx]

**Supplementary Table S4.** The CSF variables of etiologically diagnosed tubercular meningitis from Shanghai Changzheng hospital, 1999-2014

| Cases number | Gender | Age | Geographical location | Admission date | Appearance | Intracranial pressure (mm H2O) | Glucose (mmol/L) | Protein (mg/L) | Chloride (mmol/L) |
| --- | --- | --- | --- | --- | --- | --- | --- | --- | --- |
| TM Case 1 | Male | 10 | Jiangsu province | 1999.05.10 | clear and colorless | 385 | 3.2 | 2900 | 129 |
| TM Case 2 | Female | 45 | Shanghai | 2000.05.27 | clear and colorless | 210 | 2.2 | 2350 | ND |
| TM Case 3 | Male | 63 | Jiangsu province | 2001.11.14 | clear and colorless | 135 | 2.5 | 520 | 129 |
| TM Case 4 | Male | 16 | Jiangxi province | 2001.11.02 | clear and colorless | 200 | 2.2 | 1090 | 128 |
| TM Case 5 | Female | 44 | Shanghai | 2002.10.16 | clear and colorless | 205 | 4.0 | 973 | 120 |
| TM Case 6 | Female | 32 | Zhejiang province | 2002.03.25 | clear and colorless | 220 | ND | ND | ND |
| TM Case 7 | Female | 28 | Shanghai | 2002.03.05 | turbid | 280 | 2.2 | 1372 | ND |
| TM Case 8 | Male | 32 | Jiangsu province | 2002.04.26 | clear and colorless | 200 | 1.6 | 820 | 110 |
| TM Case 9 | Male | 35 | Jiangsu province | 2002.08.26 | clear and colorless | 280 | 2.5 | 1500 | 120 |
| TM Case 10 | Male | 36 | Jiangsu province | 2003.12.05 | yellow | 120 | 1.0 | 2000 | 102 |
| TM Case 11 | Female | 48 | Jiangsu province | 2003.02.21 | clear and colorless | 220 | 2.4 | 810 | 120 |
| TM Case 12 | Male | 35 | Anhui province | 2003.07.12 | turbid/yellow | 125 | 2.2 | 1900 | 107 |
| TM Case 13 | Female | 32 | Zhejiang province | 2003.07.09 | clear and colorless | 230 | 1.3 | 1690 | 115 |
| TM Case 14 | Male | 71 | Zhejiang province | 2003.08.11 | yellow | 250 | 5.3 | 1580 | ND |
| TM Case 15 | Male | 42 | Shanghai | 2004.10.22 | clear and colorless | 400 | 2.8 | 1276 | 122 |
| TM Case 16 | Male | 33 | Zhejiang province | 2004.05.13 | clear and colorless | 350 | 3.4 | 1230 | 130 |
| TM Case 17 | Male | 54 | Shanghai | 2004.08.22 | clear and colorless | 205 | ND | 1565 | ND |
| TM Case 18 | Male | 55 | Jiangsu province | 2005.10.13 | clear and colorless | ND | 2.6 | 430 | 118 |
| TM Case 19 | Male | 51 | Zhejiang province | 2005.12.23 | yellow | ND | 0.9 | 4150 | 117 |
| TM Case 20 | Male | 23 | Shanghai | 2005.12.07 | clear and colorless | 350 | 1.1 | 2050 | 108 |
| TM Case 21 | Male | 56 | Jiangsu province | 2005.03.02 | yellow | 400 | 12.6 | 845 | 111 |
| TM Case 22 | Male | 65 | Jiangsu province | 2005.04.13 | turbid/yellow | 170 | 3.4 | 1900 | 117 |
| TM Case 23 | Male | 48 | Jiangxi province | 2005.06.15 | clear and colorless | 210 | 1.4 | 1700 | ND |
| TM Case 24 | Female | 6 | Shanghai | 2005.08.20 | clear and colorless | 195 | 2.9 | 310 | 125 |
| TM Case 25 | Male | 50 | Shanghai | 2007.12.26 | clear and colorless | 400 | 0.7 | 1790 | 111 |
| TM Case 26 | Male | 52 | Jiangsu province | 2007.12.28 | turbid/yellow | 210 | 4.3 | 3000 | 103 |
| TM Case 27 | Male | 54 | Shanghai | 2007.03.09 | clear and colorless | ND | 1.3 | 1501 | 121 |
| TM Case 28 | Male | 49 | Shanghai | 2007.04.14 | clear and colorless | 250 | 2.0 | 920 | 114 |
| TM Case 29 | Male | 38 | Jiangsu province | 2007.04.22 | clear and colorless | 110 | 1.4 | 1260 | 112 |
| TM Case 30 | Male | 34 | Jiangxi province | 2007.04.23 | clear and colorless | 320 | 1.1 | 1850 | 111 |
| TM Case 31 | Female | 43 | Jiangsu province | 2007.09.25 | clear and colorless | 100 | 2.4 | 1400 | 119 |
| TM Case 32 | Male | 45 | Jiangsu province | 2008.04.10 | clear and colorless | 80 | 1.5 | 1970 | 102 |
| TM Case 33 | Male | 31 | Henan province | 2008.04.16 | clear and colorless | 230 | 2.2 | 3670 | 121 |
| TM Case 34 | Male | 57 | Zhejiang province | 2008.04.02 | clear and colorless | 240 | ND | 2593 | ND |
| TM Case 35 | Male | 69 | Jiangsu province | 2008.04.23 | yellow | ND | 1.9 | 119 | 107 |
| TM Case 36 | Male | 38 | Jiangsu province | 2008.07.18 | clear and colorless | 350 | 1.7 | 800 | 117 |
| TM Case 37 | Female | 46 | Zhejiang province | 2008.07.3 | turbid/yellow | 210 | ND | ND | ND |
| TM Case 38 | Female | 52 | Fujian province | 2008.09.22 | clear and colorless | ND | 2.7 | 1263 | ND |
| TM Case 39 | Female | 34 | Anhui province | 2008.09.28 | clear and colorless | 120 | 5.4 | 438 | 138 |
| TM Case 40 | Male | 68 | Shanghai | 2008.09.04 | clear and colorless | 100 | ND | ND | ND |
| TM Case 41 | Female | 18 | Shanghai | 2009.03.25 | clear and colorless | 170 | 3.6 | 250 | 110 |
| TM Case 42 | Female | 33 | Zhejiang province | 2010.04.29 | clear and colorless | 240 | ND | ND | ND |
| TM Case 43 | Male | 57 | Guizhou province | 2011.01.11 | clear and colorless | ND | 2.2 | 1200 | 115 |
| TM Case 44 | Male | 30 | Jiangsu province | 2011.10.15 | clear and colorless | 350 | 0.2 | 1550 | 110 |
| TM Case 45 | Male | 79 | Jiangsu province | 2011.12.03 | turbid/yellow | 350 | 2.3 | 2550 | 113 |
| TM Case 46 | Male | 42 | Jiangsu province | 2011.02.18 | clear and colorless | 130 | 0.8 | 1837 | 116 |
| TM Case 47 | Male | 46 | Jiangsu province | 2011.03.28 | clear and colorless | 235 | 3.1 | 429 | 129 |
| TM Case 48 | Male | 42 | Shanghai | 2011.04.16 | turbid/yellow | 350 | 3.5 | 840 | 96 |
| TM Case 49 | Male | 63 | Shanghai | 2011.06.13 | turbid | 175 | 2.1 | 3473 | 101 |
| TM Case 50 | Female | 31 | Anhui province | 2011.07.27 | clear and colorless | 105 | 2.1 | 1000 | 117 |
| TM Case 51 | Female | 23 | Anhui province | 2012.01.12 | yellow | 190 | 0.5 | 3039 | 123 |
| TM Case 52 | Female | 53 | Jiangsu province | 2012.11.19 | turbid | 250 | 2.1 | 1921 | 85 |
| TM Case 53 | Female | 43 | Jiangsu province | 2012.02.13 | clear and colorless | 180 | 3.5 | 950 | 119 |
| TM Case 54 | Male | 57 | Jiangsu province | 2012.03.13 | clear and colorless | 205 | 3.6 | 3753 | 117 |
| TM Case 55 | Female | 32 | Hunan province | 2012.04.24 | clear and colorless | 210 | 2.7 | 1420 | 111 |
| TM Case 56 | Female | 64 | Jiangsu province | 2012.05.04 | clear and colorless | 400 | 0.9 | 880 | 108 |
| TM Case 57 | Female | 22 | Anhui province | 2012.08.07 | turbid/yellow | 50 | 2.1 | 2120 | 106 |
| TM Case 58 | Female | 26 | Jiangsu province | 2012.09.17 | clear and colorless | 200 | 2.7 | 1060 | 111 |
| TM Case 59 | Male | 60 | Fujian province | 2013.10.31 | clear and colorless | 240 | 0.4 | 1570 | 112 |
| TM Case 60 | Female | 25 | Heilongjiang province | 2013.03.21 | clear and colorless | 160 | 1.8 | 770 | 119 |
| TM Case 61 | Male | 56 | Shanghai | 2013.09.10 | clear and colorless | 150 | 2.8 | 520 | 125 |
| TM Case 62 | Male | 57 | Jiangsu province | 2013.09.16 | clear and colorless | 350 | 0.5 | 1840 | 112 |
| TM Case 63 | Female | 17 | Jiangsu province | 2013.09.23 | clear and colorless | 350 | 2.5 | 1080 | 120 |
| TM Case 64 | Female | 57 | Jiangsu province | 2014.01.24 | yellow | 220 | 1.1 | 2594 | 111 |
| TM Case 65 | Female | 39 | Jiangsu province | 2014.02.10 | clear and colorless | 400 | 0.1 | 1000 | 111 |
| TM Case 66 | Male | 41 | Anhui province | 2014.03.20 | clear and colorless | 260 | 2.9 | 725 | 121 |
| TM Case 67 | Male | 16 | Zhejiang province | 2014.03.28 | clear and colorless | 210 | 2.5 | 1600 | 115 |
| TM Case 68 | Male | 24 | Shanghai | 2014.06.19 | clear and colorless | 240 | 1.9 | 1745 | 128 |
| TM Case 69 | Female | 33 | Shanghai | 2014.06.30 | yellow | 400 | 2.2 | 2000 | 109 |
