# Supplementary material for: Tuberculosis/cryptococcosis co-infection in China between 1965 and 2016
Source: Emerg Microbes Infect. 2017 Aug 23;6(8):e73–. doi: 10.1038/emi.2017.61 (PMC5583669; doi:10.1038/emi.2017.61)
Supplement: Supplementary Table S5 [file emi201761x7.docx]

**Supplementary Table S5**. The CSF variables of etiologically diagnosed cryptococcal meningitis from Shanghai Changzheng hospital, 1999-2014

| Cases number | Gender | Age | Geographical location | Admission date | Appearance | Intracranial pressure (mm H2O) | Glucose (mmol/L) | Protein (mg/L) | Chloride (mmol/L) |
| --- | --- | --- | --- | --- | --- | --- | --- | --- | --- |
| CM case 1 | Male | 37 | Jiangsu province | 1999.05.30 | Clear/colorless | 400 | 3.0 | 770 | 126 |
| CM case 2 | Male | 46 | Jiangsu province | 2000.06.12 | Clear/colorless | 250 | 2.5 | 590 | 122 |
| CM case 3 | Male | 21 | Jiangsu province | 2001.01.01 | Clear/colorless | 400 | 0.7 | 600 | 116 |
| CM case 4 | Male | 41 | Shanghai | 2001.06.27 | Clear/colorless | 360 | 2.8 | 380 | 116 |
| CM case 5 | Male | 42 | Jiangsu province | 2002.01.23 | Clear/colorless | 260 | 2.1 | 320 | 120 |
| CM case 6 | Female | 23 | Shanxi province | 2002.06.07 | Clear/colorless | 350 | 1.9 | 1166 | ND |
| CM case 7 | Male | 44 | Shanghai | 2002.08.27 | Clear/colorless | 150 | 2.0 | 1370 | 112 |
| CM case 8 | Male | 48 | Zhejiang province | 2002.10.16 | Yellow | 220 | 4.2 | 732 | 132 |
| CM case 9 | Female | 57 | Shandong province | 2002.11.11 | Clear/colorless | 145 | ND | 386 | ND |
| CM case 10 | Male | 15 | Jiangsu province | 2002.11.21 | Clear/colorless | 400 | 1.81 | 615 | 132 |
| CM case 11 | Female | 30 | Hubei province | 2003.01.24 | Clear/colorless | 400 | 4.52 | 100 | ND |
| CM case 12 | Female | 54 | Jiangxi province | 2003.02.11 | Clear/colorless | 450 | 3.0 | 794 | 128 |
| CM case 13 | Female | 55 | Guangxi province | 2003.03.04 | Yellow/transparent | 160 | 1.03 | 2240 | ND |
| CM case 14 | Female | 52 | Zhejiang province | 2003.08.23 | Clear/colorless | 190 | 1.2 | 3288 | 111 |
| CM case 15 | Female | 37 | Zhejiang province | 2003.11.21 | Clear/colorless | 200 | 2.9 | 510 | 112 |
| CM case 16 | Male | 20 | Shanghai | 2004.04.16 | Clear/colorless | 200 | 1.0 | 1700 | 124 |
| CM case 17 | Female | 48 | Liaoning province | 2004.05.06 | Turbid/yellow | 230 | ND | 892 | ND |
| CM case 18 | Male | 31 | Hunan province | 2005.03.08 | Clear/colorless | 270 | 2.0 | 768.2 | 123 |
| CM case 19 | Male | 24 | Shanghai | 2005.07.18 | Clear/colorless | 400 | 1.0 | 900 | 117 |
| CM case 20 | Female | 30 | Shanghai | 2005.09.05 | Clear/colorless | 400 | 7.1 | 112 | 115 |
| CM case 21 | Male | 45 | Jiangsu province | 2005.09.05 | Clear/colorless | 400 | 0.3 | 1092 | 114 |
| CM case 22 | Female | 23 | Shanghai | 2006.01.09 | Clear/colorless | 210 | 4.8 | 510 | 129 |
| CM case 23 | Male | 36 | Jiangsu province | 2006.03.10 | Clear/colorless | 150 | 0.5 | 785 | 116 |
| CM case 24 | Male | 21 | Jiangsu province | 2006.03.15 | Clear/colorless | 400 | 1.6 | 756 | ND |
| CM case 25 | Male | 48 | Hubei province | 2006.08.07 | Clear/colorless | 400 | 1.7 | 577 | 125 |
| CM case 26 | Male | 18 | Hubei province | 2006.10.23 | Clear/colorless | 270 | 1.9 | 481 | 135 |
| CM case 27 | Female | 78 | Shanghai | 2007.05.25 | Clear/colorless | 125 | ND | 1334 | ND |
| CM case 28 | Male | 49 | Shanghai | 2007.07.05 | Yellow | 450 | 3.4 | 830 | 110 |
| CM case 29 | Female | 43 | Shanghai | 2008.01.06 | Clear/colorless | 150 | 4.8 | 880 | ND |
| CM case 30 | Male | 52 | Anhui province | 2008.03.03 | Clear/colorless | 210 | 3.5 | 687 | ND |
| CM case 31 | Female | 14 | Jiangsu province | 2008.03.03 | Clear/colorless | 320 | 5.5 | ND | ND |
| CM case 32 | Female | 15 | Shanghai | 2008.04.21 | Clear/colorless | ND | 4.5 | 160 | 126 |
| CM case 33 | Male | 59 | Jiangsu province | 2008.05.12 | Clear/colorless | 330 | 2.04 | 440 | 112 |
| CM case 34 | Male | 57 | Anhui province | 2008.05.15 | Clear/colorless | 400 | 1.8 | 900 | 114 |
| CM case 35 | Male | 46 | Zhejiang province | 2008.07.25 | Clear/colorless | 250 | 2.0 | 320 | 119 |
| CM case 36 | Male | 43 | Shandong province | 2008.08.29 | Clear/colorless | 190 | 3.5 | 150 | 124 |
| CM case 37 | Female | 3 | Hunan province | 2008.09.06 | Clear/colorless | 250 | 2.7 | 279 | 124 |
| CM case 38 | Female | 31 | Anhui province | 2008.09.27 | Clear/colorless | 380 | 2.9 | 360 | 125 |
| CM case 39 | Female | 54 | Liaoning province | 2008.11.04 | Clear/colorless | 370 | 1.6 | 589 | 119 |
| CM case 40 | Male | 46 | Henan province | 2008.11.24 | Clear/colorless | 400 | 2.7 | 380 | 125 |
| CM case 41 | Female | 40 | Zhejiang province | 2009.02.01 | Clear/colorless | 400 | 2.41 | 520 | ND |
| CM case 42 | Female | 36 | Shandong province | 2009.02.12 | Clear/colorless | 270 | 2.3 | 651 | 128 |
| CM case 43 | Female | 48 | Shanghai | 2009.02.26 | Clear/colorless | 300 | 2.3 | 719 | 125 |
| CM case 44 | Male | 54 | Liaoning province | 2009.03.05 | Turbid | ND | 2.05 | 700 | 109 |
| CM case 45 | Male | 33 | Anhui province | 2009.05.04 | Clear/colorless | 210 | 1.9 | 2110 | 125 |
| CM case 46 | Female | 47 | Jiangsu province | 2009.07.22 | Clear/colorless | 280 | 2.2 | 4932 | 104 |
| CM case 47 | Female | 6 | Hunan province | 2009.07.23 | Turbid/yellow | 270 | 0.2 | 3816 | 112 |
| CM case 48 | Female | 43 | Jiangxi province | 2009.08.18 | Turbid | 400 | 0.4 | 1183 | 104 |
| CM case 49 | Female | 7 | Fujian province | 2009.12.05 | Clear/colorless | 400 | 3.17 | 140 | 112 |
| CM case 50 | Female | 41 | Jiangsu province | 2010.01.14 | Clear/colorless | 400 | 2.4 | 460 | 112 |
| CM case 51 | Male | 67 | Shanghai | 2010.02.23 | Clear/colorless | 205 | ND | ND | ND |
| CM case 52 | Female | 58 | Zhejiang province | 2010.03.11 | Clear/colorless | 80 | ND | ND | ND |
| CM case 53 | Female | 50 | Shanghai | 2010.03.26 | Yellow | 400 | 2.7 | 3110 | 121 |
| CM case 54 | Female | 27 | Hubei province | 2010.04.21 | Clear/colorless | 450 | 2.6 | 647 | 122 |
| CM case 55 | Male | 58 | Fujian province | 2010.07.20 | Clear/colorless | 350 | 4.0 | 585 | 92 |
| CM case 56 | Male | 68 | Zhejiang province | 2010.08.01 | Clear/colorless | 290 | 0.1 | 1174 | 108 |
| CM case 57 | Female | 50 | Shanghai | 2010.08.02 | Clear/colorless | 240 | 1.9 | 1691 | 108 |
| CM case 58 | Male | 43 | Jiangsu province | 2010.08.24 | Clear/colorless | 400 | 1.9 | 259 | 133 |
| CM case 59 | Female | 27 | Hubei province | 2010.10.08 | Clear/colorless | 450 | 2.6 | 647 | 122 |
| CM case 60 | Female | 34 | Anhui province | 2010.11.01 | Clear/colorless | 200 | 3.1 | 230 | 127 |
| CM case 61 | Male | 63 | Fujian province | 2010.11.24 | Clear/colorless | 230 | ND | ND | ND |
| CM case 62 | Male | 9 | Anhui province | 2011.02.21 | Clear/colorless | 400 | 4.7 | 100 | 121 |
| CM case 63 | Male | 48 | Zhejiang province | 2011.04.26 | Clear/colorless | 250 | 2.1 | 1123 | 120 |
| CM case 64 | Male | 32 | Zhejiang province | 2011.04.29 | Clear/colorless | 400 | ND | ND | ND |
| CM case 65 | Female | 45 | Zhejiang province | 2011.05.03 | Clear/colorless | 400 | 3.0 | 836 | 116 |
| CM case 66 | Female | 43 | Zhejiang province | 2011.05.16 | Clear/colorless | 160 | 2.2 | 2658 | 124 |
| CM case 67 | Male | 21 | Hubei province | 2011.06.01 | Clear/colorless | 400 | ND | ND | ND |
| CM case 68 | Male | 27 | Hubei province | 2011.07.07 | Clear/colorless | 400 | 1.9 | 1333 | 125 |
| CM case 69 | Female | 23 | Guizhou province | 2011.09.23 | Clear/colorless | 200 | ND | ND | ND |
| CM case 70 | Male | 21 | Fujian province | 2011.09.26 | Clear/colorless | 400 | 3.8 | 246 | 129 |
| CM case 71 | Female | 62 | Zhejiang province | 2011.10.05 | Clear/colorless | 400 | 1.5 | 696 | 124 |
| CM case 72 | Female | 31 | Henan province | 2011.11.03 | Clear/colorless | 340 | 3.3 | 224 | 128 |
| CM case 73 | Female | 47 | Jiangsu province | 2011.11.16 | Clear/colorless | 200 | ND | 1144 | ND |
| CM case 74 | Female | 36 | Hubei province | 2011.12.13 | Clear/colorless | 400 | 3.7 | 380 | 127 |
| CM case 75 | Male | 42 | Zhejiang province | 2012.01.14 | Clear/colorless | 300 | 3.4 | 470 | 122 |
| CM case 76 | Male | 69 | Jiangsu province | 2012.03.01 | Clear/colorless | 250 | 1.1 | 1220 | 114 |
| CM case 77 | Female | 40 | Shandong province | 2012.05.07 | Clear/colorless | 250 | 2.3 | 489 | 132 |
| CM case 78 | Male | 36 | Jiangsu province | 2012.05.08 | Clear/colorless | 150 | 1.7 | 741 | 121 |
| CM case 79 | Male | 44 | Zhejiang province | 2012.05.15 | Clear/colorless | 260 | 1.6 | 930 | 115 |
| CM case 80 | Male | 24 | Zhejiang province | 2012.05.21 | Clear/colorless | 400 | 3.5 | 316 | 126 |
| CM case 81 | Male | 42 | Zhejiang province | 2012.06.05 | Clear/colorless | 250 | 2.7 | 650 | 119 |
| CM case 82 | Female | 45 | Zhejiang province | 2012.07.20 | Clear/colorless | 400 | 1.7 | 558 | 127 |
| CM case 83 | Male | 60 | Shanghai | 2012.08.22 | Clear/colorless | 350 | 2.8 | 468 | 132 |
| CM case 84 | Male | 31 | Liaoning province | 2012.10.29 | Clear/colorless | 205 | 2.8 | 670 | 131 |
| CM case 85 | Female | 50 | Shanghai | 2012.11.20 | Turbid | 380 | 1.3 | 617 | 118 |
| CM case 86 | Female | 40 | Hubei province | 2013.03.08 | Turbid | 120 | 2.0 | 457 | 126 |
| CM case 87 | Male | 43 | Hunan province | 2013.03.14 | Clear/colorless | 400 | 1.6 | 1520 | 113 |
| CM case 88 | Male | 27 | Jiangsu province | 2013.03.23 | Clear/colorless | 400 | 2.6 | 354 | 123 |
| CM case 89 | Male | 31 | Jiangxi province | 2013.03.27 | Clear/colorless | 170 | 3.2 | 410 | 116 |
| CM case 90 | Male | 56 | Zhejiang province | 2013.05.29 | Clear/colorless | 250 | 2.5 | 281 | 126 |
| CM case 91 | Male | 52 | Shanghai | 2013.06.14 | Clear/colorless | 400 | 1.6 | 2734 | 105 |
| CM case 92 | Female | 45 | Henan province | 2013.07.09 | Clear/colorless | 290 | 3.8 | 549 | 115 |
| CM case 93 | Male | 40 | Hubei province | 2013.07.26 | Turbid/yellow | 90 | 0.2 | 2300 | 90 |
| CM case 94 | Male | 43 | Hunan province | 2013.09.18 | Clear/colorless | 310 | 2.2 | 1093 | 128 |
| CM case 95 | Male | 22 | Hebei province | 2013.09.23 | Clear/colorless | 250 | ND | 280 | ND |
| CM case 96 | Male | 23 | Jiangxi province | 2013.10.09 | Clear/colorless | 195 | 3.3 | 1744 | 172 |
| CM case 97 | Male | 31 | Shanghai | 2013.10.10 | Clear/colorless | 400 | 0.4 | 782 | 121 |
| CM case 98 | Male | 59 | Jiangxi province | 2013.10.11 | Turbid | 220 | 2.6 | 1007 | 106 |
| CM case 99 | Female | 50 | Shanghai | 2013.11.20 | Clear/colorless | 380 | 5.6 | 367 | 129 |
| CM case 100 | Male | 20 | Jiangsu province | 2013.11.20 | Clear/colorless | 310 | 3.0 | 321 | 126 |
| CM case 101 | Male | 12 | Zhejiang province | 2013.12.11 | Clear/colorless | 400 | 2.5 | 532 | 160 |
| CM case 102 | Male | 24 | Shanghai | 2013.12.27 | Clear/colorless | 400 | 1.6 | 775 | 126 |
| CM case 103 | Male | 51 | Jiangxi province | 2013.12.6 | Clear/colorless | 400 | 2.2 | 290 | 109 |
| CM case 104 | Male | 49 | Hubei province | 2014.01.22 | Clear/colorless | 400 | 0.3 | 1546 | 134 |
| CM case 105 | Male | 43 | Shanxi province | 2014.02.07 | Clear/colorless | 140 | 0.2 | 310 | 116 |
| CM case 106 | Male | 31 | Jiangsu province | 2014.02.12 | Clear/colorless | 400 | 1.8 | 836 | 124 |
| CM case 107 | Male | 42 | Shanxi province | 2014.03.05 | Clear/colorless | 220 | 1.0 | 711 | 129 |
| CM case 108 | Female | 19 | Sichuan province | 2014.03.13 | Clear/colorless | 340 | 0.4 | 914 | 134 |
| CM case 109 | Female | 55 | Jiangxi province | 2014.08.06 | Clear/colorless | 400 | 5.8 | 365 | 132 |
| CM case 110 | Female | 50 | Anhui province | 2014.08.14 | Clear/colorless | 400 | 2.5 | 343 | 130 |
| CM case 111 | Male | 45 | Shanghai | 2014.08.15 | Clear/colorless | 110 | 4.3 | 205 | 128 |
