# Supplementary material for: Tuberculosis/cryptococcosis co-infection in China between 1965 and 2016
Source: Emerg Microbes Infect. 2017 Aug 23;6(8):e73–. doi: 10.1038/emi.2017.61 (PMC5583669; doi:10.1038/emi.2017.61)
Supplement: Supplementary Table S6 [file emi201761x8.docx]

**Supplementary Table S6**. Univariate analysis comparing the CSF variables among all patients (including the etiologically diagnosed) of tubercular/cryptococcal meningitis, tubercular meningitis, and cryptococcal meningitis

| CSF parameters | tubercular/cryptococcal meningitis  Mean (95% CI) | tubercular meningitis  Mean (95% CI) | Cryptococcal meningitis  Mean (95% CI) | *P* value |
| --- | --- | --- | --- | --- |
| Intracranial pressure (mmH_2_O) | 333.6±31.3 [266.9, 400.3] n=16 | 235.4 ±11.7 [212.0, 258.8] n=63 | 303.3±9.6 [284.2, 322.3] n=109 | <0.05^a^; >0.05^b^; <0.05^c^ |
| CSF-glucose (mmol/L) | 1.55±0.19 [1.16, 1.95] n=26 | 2.4 ± 0.2 [1.9, 2.8] n=63 | 2.4±0.1[2.2, 2.7] n=100 | <0.05^a^; <0.05^b^; >0.05^c^ |
| CSF-protein (mg/L) | 1620.1±281.9 [1035.6, 2204.8] n=23 | 1557.7±109.8 [1338.4, 1777.0] n= 65 | 867.4±78.6 [711.6, 1023.2] n=104 | >0.05^a^; <0.05^b^; <0.05^c^ |
| CSF-chloride (mmol/L) | 114.6±4.8 [104.6, 124.5] n=24 | 115.1±1.2 [112.7, 117.5] n=58 | 121.5±1.2 [119.2, 123.8] n=92 | >0.05^a^; <0.05^b^; <0.05^c^ |

Note: a: tubercular/cryptococcal meningitis vs. tubercular meningitis; b: tubercular/cryptococcal meningitis vs. cryptococcal meningitis; c: tubercular meningitis vs. cryptococcal meningitis
