# Supplementary material for: Tuberculosis/cryptococcosis co-infection in China between 1965 and 2016
Source: Emerg Microbes Infect. 2017 Aug 23;6(8):e73–. doi: 10.1038/emi.2017.61 (PMC5583669; doi:10.1038/emi.2017.61)
Supplement: Supplementary Table S7 [file emi201761x9.docx]

**Supplementary Table S7**: Receiver operating characteristic (ROC) curve area comparison between intracranial pressure, glucose, protein, chlorine, and the combined use of above values

| Pair | Area Difference | Standard Error | 95% Confidence Interval | Z Statistic | P Value |
| --- | --- | --- | --- | --- | --- |
| Intracranial pressure, Glucose | -0.03 | 0.13 | -0.28 to 0.22 | -0.26 | 0.80 |
| Intracranial pressure, Protein | -0.05 | 0.13 | -0.31 to 0.21 | -0.38 | 0.70 |
| Intracranial pressure, Chlorine | -0.10 | 0.13 | -0.35 to 0.16 | -0.73 | 0.46 |
| Intracranial pressure, Combined | -0.24 | 0.12 | -0.48 to -0.01 | -2.02 | 0.04 |
| Glucose, Protein | -0.02 | 0.10 | -0.22 to 0.18 | -0.18 | 0.86 |
| Glucose, Chlorine | -0.06 | 0.10 | -0.26 to 0.13 | -0.64 | 0.52 |
| Glucose, Combined | -0.21 | 0.08 | -0.37 to -0.04 | -2.49 | 0.01 |
| Protein, Chlorine | -0.05 | 0.11 | -0.25 to 0.16 | -0.42 | 0.67 |
| Protein, Combined | -0.19 | 0.09 | -0.37 to -0.01 | -2.06 | 0.04 |
| Chlorine, Combined | -0.15 | 0.09 | -0.32 to 0.028 | -1.64 | 0.10 |
