# Supplementary material for: Tuberculosis/cryptococcosis co-infection in China between 1965 and 2016
Source: Emerg Microbes Infect. 2017 Aug 23;6(8):e73–. doi: 10.1038/emi.2017.61 (PMC5583669; doi:10.1038/emi.2017.61)
Supplement: Supplementary Table S8 [file emi201761x10.docx]

**Supplementary Table S8**. Therapeutic details of TB/cryptococcosis co-infection

| Study number | HIV-infected | Affected site of the co-infection | Anti-TB treatment | Antifungal treatment |
| --- | --- | --- | --- | --- |
| 1 | No | Cryptococcosis (Brain) + TB (Lung) | INH+ PAS+ SM | ND |
| 2 | No | Cryptococcosis (Brain) + TB (Brain+Lung) | Died before treating | ND |
| 3 | No | Cryptococcosis (Brain) + TB (Brain) | INH+ PAS+ SM+ EMB | AmB + 5-FC |
| 3 | No | Cryptococcosis (Brain) + TB (Brain) | INH+ PAS+ SM+ EMB | AmB + 5-FC |
| 3 | No | Cryptococcosis (Brain) + TB (Brain) | INH+ SM+ EMB+ PZA | AmB + 5-FC |
| 4 | No | Cryptococcosis (Brain) + TB (Brain+Lung) | ND | ND |
| 5 | No | Cryptococcosis (Brain) + TB (Brain) | ND | ND |
| 6 | No | Cryptococcosis (Brain) + TB (Brain+Lung) | INH + SM + PZA | ND |
| 7 | No | Cryptococcosis (Brain) + TB (Brain) | INH+ RIF+SM | FCZ + 5-FC |
| 8 | No | Cryptococcosis (Brain) + TB (Brain) | ND | ND |
| 8 | No | Cryptococcosis (Brain) + TB (Brain) | ND | ND |
| 9 | ND | Cryptococcosis (Brain) + TB (Lung) | ND | ND |
| 10 | No | Cryptococcosis (Brain) + TB (Brain) | INH + RIF + EMB + SM | FCZ |
| 11 | No | Cryptococcosis (Brain) + TB (Lung) | INH+ RFP+ PZA+ SM | give up treatment |
| 12 | No | Cryptococcosis (Brain) + TB (Lung) | ND | ND |
| 13 | No | Cryptococcosis (Brain) + TB (Brain) | ND | FCZ |
| 14 | No | Cryptococcosis (Brain) + TB (Lung and bone) | INH+ RIF+ SM+EMB | FCZ |
| 14 | No | Cryptococcosis (Brain) + TB (Brain) | INH+ RIF+ EMB+ Ofloxacin | FCZ |
| 15 | No | Cryptococcosis (Brain) + TB (Brain) | INH+ PAS+ RIF+ PZA+ SM | FCZ |
| 16 | No | Cryptococcosis (Brain) + TB (Brain+Lung) | INH+ RIF+ SM+ PZA | AmB + FCZ |
| 17 | No | Cryptococcosis (Blood) + TB (Liver) | ND | AmB + FCZ + 5-FC |
| 18 | ND | Cryptococcosis (Brain) + TB (Lung) | ND | ND |
| 19 | No | Cryptococcosis (Brain) + TB (Lung) | ND | FCZ |
| 20 | Pos | Cryptococcosis (Brain) + TB (Brain) | RIF+ INH+ PZA+ PAS | FCZ |
| 21 | No | Cryptococcosis (Brain) + TB (Brain+Lung) | INH+ RIF+ PZA | AmB + Intrathecal injection of AmB + FCZ |
| 22 | No | Cryptococcosis (Brain) + TB (Brain) | INH+EMB+RIF+PZA | AmB + FCZ + 5-FC |
|  | No | Cryptococcosis (Brain) + TB (Brain+Lung) | INH+EMB+RIF+PZA | AmB |
|  | No | Cryptococcosis (Brain) + TB (Brain+Lung) | INH+EMB+RIF+PZA | AmB + FCZ |
| 23 | No | Cryptococcosis (Brain) + TB (Brain) | INH+ RIF+ PZA | AmB + FCZ |
| 24 | No | Cryptococcosis (Brain) + TB (Brain) | INH+ RFP+ PZA+ EMB+ SM | AmB + FCZ + 5-FC |
| 25 | ND | Cryptococcosis (Lung) + TB (Lung) | INH+ RFP+ PZA+ EMB+ SM | ND |
| 26 | ND | Cryptococcosis (Lung) + TB (Lung) (N=2)；  Cryptococcosis (Brain) + TB (Lung)；  Cryptococcosis (Brain) + TB (disseminated) | ND | ND |
| 27 | No | Cryptococcosis (Brain) + TB (Brain) | INH+ RIF+ PZA+ EMB+ PAS | AmB + FCZ |
| 28 | No | Cryptococcosis (Lung) + TB (Lung) | ND | ND |
| 29 | No | Cryptococcosis (Blood+Skin) + TB (Blood) | Died before Anti-TB treatment | FCZ |
| 30 | No | Cryptococcosis (Brain) + TB (Brain) | ND | AmB + FCZ + 5-FC |
| 31 | No | Cryptococcosis (Brain) + TB (Brain) | INH+ EMB | AmB + FCZ + 5-FC |
|  | No | Cryptococcosis (Brain) + TB (Brain) | INH+ RFP+ PZA+ EMB | AmB + FCZ + 5-FC |
|  | No | Cryptococcosis (Brain) + TB (Brain) | INH+ RFP+ PZA+ EMB | AmB + FCZ + 5-FC+ Allicin |
|  | No | Cryptococcosis (Brain) + TB (Brain) | INH+ RFP+ PZA+ EMB | AmB + FCZ + 5-FC |
|  | No | Cryptococcosis (Brain) + TB (Brain) | INH+ RFP+ PZA+ EMB | AmB + FCZ + 5-FC+ Allicin |
| 32 | No | Cryptococcosis (Lung+Skin)+ TB (Lung) | INH+ RIF+ EMB+ PZA | FCZ |
| 33 | Pos | Cryptococcosis (Brain) + TB (Brain) | INH+ SM+ RIF+ PZA | AmB + 5-FC |
| 34 | No | Cryptococcosis (Brain) + TB (Brain +Lung) | INH+ RIF+ PZA+EMB | FCZ |
| 34 | No | Cryptococcosis (Brain) + TB (Brain +Lung) | RIF+ PZA+ EMB+ INH | AmB + FCZ + 5-FC |
| 35 | No | Cryptococcosis (Lung) + TB (Lung) | ND | AmB + FCZ |
| 36 | Pos (n=6) | 6Cryptococcosis (Brain) + TB (Lung) | ND | AmB + FCZ |
| 37 | No | Cryptococcosis (Brain) + TB (Brain) | INH+ RFP+ PZA+ EMB+ SM | FCZ+ Allicin |
| 38 | Pos (n=11) | Cryptococcosis (Lung) + TB (Lung) | INH+ RFP+ PZA+ EMB | AmB + FCZ |
| 39 | No | ND | ND | AmB (n=11) or FCZ (n=12) |
| 40 | No | Cryptococcosis (Brain) + TB (Brain) | INH+ PAS+ RIF+ PZA+ EMB | Liposome AmB + 5-FC |
| 41 | No | Cryptococcosis (Brain) + TB (Lung) | INH+ RIF+ EMB+ PZA | AmB + 5-FC |
| 42 | Pos | Cryptococcosis (Brain) + TB (Lung) | INH+ RIF+ EMB+ PZA | Liposome AmB + 5-FC |
| 43 | ND | Cryptococcosis (Brain) + TB (Brain) | INH+ RFP+ PZA+ EMB | AmB (n=26) or FCZ (n=26) |
| 44 | ND | Cryptococcosis (Lung) + TB (Lung) | ND | ND |
| 45 | No | Cryptococcosis (Brain) + TB (Brain) | INH+ RFP+ PZA+ EMB | ND |
| 46 | No | Cryptococcosis (adrenal gland) + TB (Lung) | ND | FCZ |
| 47 | No | Cryptococcosis (Lung) + TB (Lung) | INH+ RFP+ PZA+ EMB | FCZ |
| 48 | ND | Cryptococcosis (Lung) + TB (Lung) | ND | ND |
| 49 | ND | Cryptococcosis (Lung) + TB (Lung) | ND | ND |
| 50 | Pos | Cryptococcosis (Brain) + TB (Lung) | ND | ND |
| 51 | No | Cryptococcosis (Lung) + TB (Lung) | PZA+ RIF+ INH+ EMB | FCZ |
| 52 | Pos (n=1) | Cryptococcosis (Brain) + TB (Brain) | INH+ RFP+ EMB | FCZ |
| 53 | No | Cryptococcosis (Lung) + TB (neck lymph node) | ND | FCZ |
| 54 | Pos (n=4) | Cryptococcosis (Brain) + TB (Lung) | ND | ND |
| 55 | No | Cryptococcosis (Lung) + TB (Lung) | INH+ RFP+ PZA+ EMB | FCZ |
| 56 | No | Cryptococcosis (Lung) + TB (Lung) | INH+ RFP+ PZA+ EMB+ SM | ND |
